# Feral Cat Responses to Silver Vine: Implications for Feral Cat Management

**DOI:** 10.1007/s10886-026-01707-5

**Published:** 2026-04-13

**Authors:** Natasha E. Tay, Bob Du, Todd A. Gillam, Melissa L. Thomas, Dave Algar, Patricia A. Fleming

**Affiliations:** 1 Harry Butler Institute, Murdoch University, 90 South Street, Murdoch, WA 6150 Australia; 2 School of Agricultural Sciences, Murdoch University, 90 South Street, Murdoch, WA 6150 Australia; 3 School of Mathematics, Statistics, Chemistry and Physics, Murdoch University, WA 6150 90 South Street, Murdoch, Australia; 4 Department of Biodiversity, Conservation and Attractions, Locked Bag 104, Bentley Delivery Centre, WA 6983 Bentley, Australia; 5 Environmental and Conservation Sciences, Murdoch University, 90 South Street, Murdoch, WA 6150 Australia

**Keywords:** *Actinidia polygama*, Actinidine, Catnip, Iridomyrmecin, Matatabi, Nepetalactone

## Abstract

Lures are an important tool used for pest management of invasive and feral species. The euphoric ‘catnip’ response in the domestic cat (*Felis catus*) is triggered by volatile organic compounds (VOCs) produced by several plants. These compounds elicit sniffing, licking, chewing, and rubbing responses in cats, which could increase the efficacy of various management tools for feral cats. We examined feral cat behavioural responses to four commercial plant products that are purported to elicit the ‘catnip’ response—Fishmint (a mix of five plant species) and three silver vine (*Actinidia polygama*) products. The behavioural responses of 12 feral cats, individually exposed to each of the products in a controlled environmental setting, were recorded. There were significant differences in the amount of time cats spent interacting with the plant materials, with marked differences in intensity of their interactions. Chemical analysis of the plant products using Gas Chromatography and Mass Spectrometry were carried out using headspace and solvent extraction. The silver vine plant material that they appeared most strongly interested in had the highest concentration of actinidine present, but nepetalactone, isodihydronepetalactone (and isomers), and iridomyrmecin were also evident in all the silver vine samples. Silver vine products elicited behavioural responses from feral cats, indicating their potential as effective lures. Such lures could enhance detection and monitoring in feral cat management programs. We report chemical differences in plant lure compounds, which likely reflect the plant part used, and/or processing and handling of plant material, and raise considerations for manufacture and presentation of cat lures.

**Supplementary Information:**

The online version contains supplementary material available at 10.1007/s10886-026-01707-5.

## Introduction

Lures are widely used in the management of invasive and pest species to aid in their detection, monitoring and control (e.g., El-Sayed et al. 2009). They function by attracting the target species through a variety of signals, including visual (e.g., Davis et al. 2015; McLean et al. 2017), auditory (e.g., Schwarzkopf and Alford 2007), or chemical cues such as food sources (e.g., Clapperton et al. 1994; Sabier et al. 2022) or sex pheromones (e.g., Saveer et al. 2023; Walgenbach et al. 2021). Lures have proven valuable in the early detection of invasive or pest species before populations become established (e.g., Cha et al. 2018; Hartshorn et al. 2021), in the monitoring of established populations (e.g., Sharov et al. 2002), and in population control — where individuals are attracted to lethal traps or treated baits (e.g., El-Sayed et al. 2009; Wayne et al. 2024). However, the attraction of non-target species to lures can influence lure performance through competition with target species for lure access, and can lead to damage or depletion of the lure.

An effective approach to improving lure performance and reduce non-target attraction is to harness chemical signals that elicit innate, species-specific behavioural responses. For example, the heritable euphoric catnip or matatabi response in cats (Felidae family) (Todd 1962,1963) is linked to volatile compounds produced by several plants (Table 1 and references therein). While the chemical composition of these plants varies, the catnip response has been linked with bioactive iridoid compounds — nepetalactone, nepetalactol, iridomyrmecin, actinidine, and derivatives or isomers of these compounds (Table 1) (Tucker and Tucker 1988). For example, Uenoyama et al. (2021) demonstrated that the iridoid nepetalactol is the major active component of silver vine (also known as matatabi or orange kiwifruit; *Actinidia polygama*), with a synthesised version of this compound increasing plasma β-endorphin levels in cats and eliciting the classic rubbing response in domestic cats (*Felis catus*), while pharmacological inhibition of µ-opioid receptors suppressed the response. Cats perceive these secondary plant metabolites through smell, while oral administration of active compounds induces no response (Todd 1963; Uenoyama et al. 2021). The euphoric effects start immediately after exposure but only last for about 5–15 min, followed by a refractory period of non-responsiveness of several minutes (Todd 1963).

**Table 1:** Plant materials and their chemicals that have been reported to elicit a ‘euphoric response’ in domestic cats (*Felis catus*)

		Chemistry						Cat behavioural responses			
Plant material		Nepetalactol	Nepetalactone	Isodihydronepetalactone (and isomers)	Iridomyrmecin	Actinidine	Ref.	Plant part (where specifically indicated)	Proportion of cats that responded		Ref.
Catnip *Nepeta cataria* L. (Lamiales: Lamiaceae)			+++			+	[2]	leaves and flowers	68%		[2]
			+++				[4]		38%		[3]
			+				[1]		69%		[5]
									100% (20% showed ‘active responses’)		[6]
*Nepeta* spp.			+++				[1, 4]				
Silver vine/matatabi *Actinidia polygama* Maxim (Ericales: Actinidiaceae)		+++		+	++		[7]		57%		[7]
		+++			+	++	[1]	fruit galls	79%		[2]
			+++		+++	+++	[4]				
			+	+	+	+++	[8]	fruit galls	C 80%		[8]
			+	+	+	+++	[8]		J 70%		[8]
			+++	+	+++	++	[8]		A 73%**		[8]
				+++	++	++	[2]				
Tatarian honeysuckle *Lonicera tatarica*L. (Dipsacales: Caprifoliaceae)			+			++	[2]	wood and sawdust	53%		[2]
Valerian *Valeriana officinalis *L. (Dipsacales: Caprifoliaceae)					+	++	[2]	root	47%		
						+++	[4]				
Indian nettle *Acalypha indica *L. (Malpighiales: Euphorbiaceae)			+		+		[9]	root	67%		[10]
								root	66%		[3]

Semi-quantitative scoring: + low (bottom quartile), ++ medium (25-75% range) and +++ high (top quartile) concentrations, relative to other plant materials analysed in the same study

** Mix of catnip and silver vine

References: 1. Keesey, et al. (2019); 2. Bol, et al. (2017); 3. Wickramaratne, et al. (2022); 4. Tucker and Tucker (1988) review ; 5. Todd (1962); 6. Espín-Iturbe, et al. (2017); 7. Uenoyama, et al. (2021); 8. present study; 9. Scaffidi, et al. (2016); 10. Rupasinghe, et al. (2022)

The euphoric response is evident in pet cats as a sequence of playful interactive behaviour, escalating from (1) attentiveness, sniffing, (2) salivation, licking and chewing with head shaking, (3) chin and cheek rubbing, through to (4) head-over rolling and body rubbing (Todd 1962; Yoshii et al. 1963). Cats will sometimes also drool and hold or paw at the plant materials with hind feet (Todd 1962). Because of the euphoric response elicited, many of these plant materials are promoted as a method for behavioural enrichment of pet cats (Ellis et al. 2022) and sold widely by retailers for this purpose (e.g., https://www.whatcatsneed.com/cat/silvervine). For example, silver vine has been studied for its positive impact on the behaviour and welfare of captive cats: Myatt (2014) reported that silver vine had a significant effect on the frequency of sitting and playing in cats, while Zhang and McGlone (2020) found that adding silver vine to upright scratchers increased the duration and frequency of interactions by adult cats.

Among the plants known to elicit the catnip-like behavioural response (Table 1), silver vine stands out for its particularly strong effects, with a comparative study finding that cats respond most intensely to silver vine (Bol et al. 2017). Silver vine has long been recognised as a cat stimulant (Fairchild 1906), particularly in Japan (Bol et al. 2017). Cat responses to silver vine were originally reported in the scientific literature in 1906, after imported seeds from China growing in an arboretum were ravaged by the neighbourhood’s cats (Fairchild 1906). However, a century after its original report, Abramson et al. (2012) noted that there remains few published studies regarding the biological and behavioural impact of silver vine. Beyond its value for enrichment and welfare, silver vine’s plant derived iridoid compounds show promise for improving the efficacy of feral cat control methods, warranting further investigation.

As part of feral cat management programs in Australia, lures are commonly used to increase camera trap detections (e.g., Lohr et al. 2024). However, despite trials with different lure types, including artificial olfactory attractants, food-based lures, and visual stimuli, results have generally been inconsistent or only marginally effective in attracting feral cats (e.g., Paton et al. 2024; Read et al. 2015). This highlights the need for more reliable lures that can elicit strong and consistent behavioural responses across individuals. The galled fruit of silver vine has proven effective on cats that are not receptive to catnip (Bol et al. 2017), and therefore could have broader applicability as a potential lure for feral cats. Some authors have suggested that strength of behavioural response increases with the age of the cat (behavioural responses of juveniles and adults differ, Espín-Iturbe et al. 2017), but the animal’s sex or personality appear to have little impact on responses (Bol et al. 2017; Espín-Iturbe et al. 2017; Todd 1963). *Actinidia polygama* lure could therefore have an advantage over social lures (e.g., urine or faeces of scents of conspecifics) or food lures, which can bias which cohorts of animals in the population respond.

The objectives of this experiment were to (1) screen four plant materials, including three commercially available dried silver vine products, to identify which was the most attractive to cats, and (2) use gas chromatography mass spectrometry (GC-MS) to identify the presence of key known bioactive ingredients in the commercial products.

## Methods and Materials

### **Cat Capture and Housing**

This project was carried out under a Department of Biodiversity, Conservation and Attraction (DBCA) animal ethics permit (2021–13 F) and under reciprocal agreement with Murdoch University Animal Ethics Committee (N3355/21). Twelve cats were used in this trial (6 males 3.49 ± 1.48 kg, 6 females 3.34 ± 0.58 kg). Unowned intact cats were captured from either the Wagin Rubbish Tip on the 13 July 2023 (cat numbers 2–10) or at the Seabird Rubbish Tip on 20 July 2023 (cat numbers 1,11,12) in Western Australia, Australia (Table 2). 

**Table 2:** Details of cats used in the trials and the number of treatment trials they participated in. NA indicates cat was removed from the trial. The three silver vine products were sourced from China (C), Japan (J) and the USA (A)

Cat ID	Sex	Body mass	Date captured	Source	Control	Fishmint	Silver vine-C	Silver vine-J	Silver vine-A
A	M	5.2 kg	20/07/2023	Seabird	2	2	2	2	2
B†	M	2.4 kg	13/07/2023	Wagin	1	NA	NA	NA	1
C	M	3.1 kg	13/07/2023	Wagin	2	2	2	2	2
D‡	F	3.8 kg	13/07/2023	Wagin	1	1	1	1	1
E	F	3.1 kg	13/07/2023	Wagin	2	2	2	2	2
F	F	3.4 kg	13/07/2023	Wagin	2	2	2	2	2
G	F	2.7 kg	13/07/2023	Wagin	2	2	2	2	2
H	F	2.6 kg	13/07/2023	Wagin	2	2	2	2	2
I	M	1 kg	13/07/2023	Wagin	2	2	2	2	2
J	F	4 kg	13/07/2023	Wagin	2	2	2	2	2
K†	M	4.1 kg	20/07/2023	Seabird	1	2	2	2	1
L‡	M	5 kg	20/07/2023	Seabird	1	1	1	1	1

† cat ‘B’ was replaced by cat ‘K’ after 2 trials

‡ cat ‘D’ was replaced with cat ‘L’ for the November trials

Twenty-five Sheffield wire cage traps (60 × 20 × 20 cm) with treadle plates (Sheffield Wire Products, Welshpool Western Australia) were deployed and these were baited with chicken (KFC) cable tied to the back of the trap. Traps with a canvas cover over the top were placed in sheltered locations; only open at night; and collected at sunrise the following morning. The traps were operational for one night only. It took < 1 h to complete trap checking and initial processing. All captured cats were inspected to assess possible injury, ownership (i.e., PIT tag) and overall health condition. Cats were transported in their cages, in an enclosed vehicle, to the Wildlife Research Centre, Woodvale, Western Australia, on the morning of capture.

The cats were used for a series of non-invasive behavioural trials during the period that they were held (only two of these trials are described in the present study). We have no estimate for the age of these unowned cats; one individual was likely sub-adult at first capture (1 kg), but the remaining individuals were adult (≥ 2.6 kg) (Table 2). Cats were held at the Wildlife Research Centre in purpose-built pens each fitted with a remote video monitoring system, thus minimising disturbance throughout the trials. Cats were housed individually in outdoor pens with concrete floor measuring 1.0 × 3.0 × 2.3 m (W x L x H, see **Supplementary Figure **S1). Ten pens were arranged side by side with shared wire mesh walls. All pens were in partial shade throughout the day. Shelter consisted of a wooden kennel (approximately 57 × 85 × 59 cm), each containing a hessian sack to provide bedding. Each pen contained a litter tray and enrichment items, including branches for scratching and climbing, and PVC pipes for hiding. Tinned cat food (half a can ~ 200 g) and dried cat biscuits (handful ~ 30 g) were supplied daily to each animal, and water was available ad libitum. Human interaction was restricted to daily pen cleaning and monitoring of the animals’ general well-being (such as appearance, gait, and food intake), while trying to minimise disturbance. Animals were held for two weeks to acclimatise to the environment and husbandry procedures prior to trials commencing. 

### Experimental Design

Four plant products (lure treatments) were tested. (1) For an olfactory control, we included dried ‘Fishmint’ powder (Traditional Chinese Medicine CN-Z20010131, Guangxi Bangqi Pharmaceutical Co., Ltd., Guangxi, China), which contains five main plant ingredients (*Houttuynia cordata, Scutellaria baicalensis, Isatis indigotica, Forsythia suspensa, Lonicera japonica*). The Fishmint product was packaged in pouches each containing ~ 6 g of brownish granules that had a sweet taste, slight bitterness, and a ‘fishy’ smell. Three silver vine products were purchased from commercial suppliers: (2) Silver vine-C: Catwant® Silver vine Gall Fruits Powder (Heilongjiang, China, packed in Liaoning, China, sold by Guangzhou Performance Pet Suppliers, China, expiry 24 months), shipped as 10 g doses in glass vials (Alibaba); (3) Silver vine-J: Smack Silver vine for Cat (Nagoya, Japan), shipped as 0.5 g doses in foil packaging (Amazon); and (4) Silver vine-A: Meowy Janes All Natural Magic Cat Play Powder – catnip and silver vine blend (Meowy Janes, New Jersey, USA), shipped in a single 45 g tin (Amazon).

Lures were prepared by a single researcher (NT) in a sterile laboratory environment and were powdered and weighed (0.5 g individual aliquots) into individual Eppendorfs for storage and to minimise the need to handle the powder on the day of presentation. Bol et al. (2017) demonstrated that 0.5 g to 1 g of silver vine powder was sufficient to elicit a response in domestic cats. The lures were presented on a 130 × 90 mm scourer pad (‘lure-presenter’) attached to a paving brick with elastic bands to prevent it being moved by the wind or the cat, with the bricks placed approximately 2 m away from the kennel shelter. The bricks were left in the pens for the entire duration of the trial, while new scourer pads were fitted under the elastic bands for each trial (generally by one researcher, wearing gloves). For each presentation, 0.5 g of a lure treatment was tipped out and spread onto the scourer pad. This presentation allowed the cat direct exposure to the lure while minimising the opportunity for cats to simply ingest the material. In addition to the four lure products, we also presented cats with a control, which consisted of the same lure-presenter, but without a lure.

Each cat was tested with each of the four lure treatments and one control, with the exceptions noted in Table 2. Cats were exposed to only one treatment or control at a time, and lure types were rotated between trials (see **Supplementary Table**S2). Cats were housed individually in side-by-side pens, but only every second penned cat was used on any given day to reduce the potential for contamination due to proximity of the neighbouring pen. Cats were rested for at least one day between trials.

A total of 100 trials were scored; 50 trials were carried out for 11 cats between 21 August to 2 September 2023, and 50 trials were carried out on 10 cats between 6 and 24 November 2023 (Table 2). The timing of all trials coincided with the cat’s feeding time. The August trials were completed at the beginning of the day, while the November trials were completed at the end of the day, with feeding time shifted to the end of the day to accommodate trial timing. The trials were repeated because – upon analysis of the August data – it was clear that there was a large proportion of cats that were inactive at the beginning of the day, and so the trial was repeated at a time that the cats were more interactive with their environment. All lures were presented to each cat in both trial sessions (except where cats were replaced, as indicated in Table 2). For each trial, the lure treatments were placed in the pens for ~ 2 h prior to feeding time, and the treatments were removed at feeding (i.e., trial lengths were ~ 2 h in length).

### Video Analyses

Videos were scored by one observer (NT) blinded to the experimental treatment that each cat had received. The behaviour ethogram was based on Wickramaratne et al. (2022) (Table 3). The ethogram was coded into behavioural observation research interactive software (BORIS® version 8.20.7) (Friard and Gamba 2016) by assigning a code to each of the behaviour categories contained in the ethogram (Table 3). Behaviour was then logged for each video clip played on BORIS, with individual cats as subjects and lure type as the independent variable. Video recordings of each cat averaged 1.57 ± 0.40 h per trial (range 0.8–2 h; shorter times were due to logistical constraints with staff access over the duration of the 100 trials). Recordings of each trial began when personnel left the pen area (‘start’) following placement of the lures and ended either when personnel returned to feed the cats (‘end’) or at the end of the video. As cats often retreated to their shelters when personnel were in the vicinity of the pens, we considered the ‘active duration’ of each trial to be when the cat first emerged from its shelter after personnel had left the pen area till the end of the recording. A potential interaction zone (‘zone’) was defined as the length of the brick (240 mm; approximately half a body length for the cats), and cats were described as being ‘within the interaction zone’ when their head was closer than this to the lure-presenter (i.e., they were in close enough proximity to likely detect the lure). Interactions were scored as (1) sniffing, (2) oral (licking, chewing) or (3) body interactions (holding, scratching and head rubbing) (Table 3). We carried out a Cochran Q test for cat response (responded = 1, or did not respond = 0) followed by McNemar pairwise post-hoc tests (with Bonferroni correction). Behavioural data were then exported and analysed in R ver. 4.1.2 (R Core Team 2018) for further analysis.

### Data Analysis

**Fig. 1:**
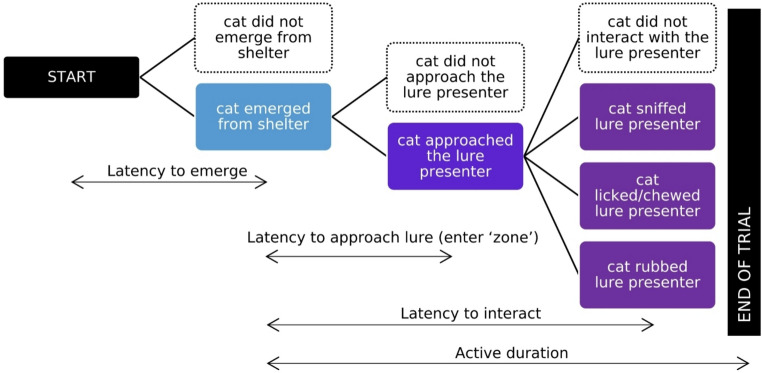
Schematic of the methods for behavioural testing of cats

For each trial (Fig. 1), we calculated the animal’s

- latency to emerge from the shelter (seconds) = emergence time – trial start time.
- active duration (hours) = time between emergence to the end of the video.
- latency to approach within half a body length of the lure or ‘enter zone’ (seconds) = time when first within half a body length of the lure – emergence time.
- total amount of time in the ‘lure zone’ (seconds) = total amount of time in the zone.
- latency to lure interaction (seconds) = initial interaction time – emergence time.
- total amount of time interacting (seconds) = sum of all time spent sniffing, oral and body interactions.

**Table 3:** Behaviour ethogram of cats, following Wickramaratne et al. (2022)

Type	Category	Behaviour	‘Intensity’ score	Description
Single event	Timepoint	Start		After lure was introduced and personnel have left the cat pens and were no longer heard/seen.
Single event	Timepoint	Emergence		Cat first emerged from its shelter during the trial.
Time period	Potential interaction zone	Zone		Cat’s head was within half a body length of the lure-presenter.
Time period	Interaction (sniffing)	Sniffing	1	Nasal investigation while focusing on the lure-presenter.
Time period	Interaction (oral)	Chewing	2	Grinding or comminution the lure-presenter or lure using teeth, while exhibiting jaw and/or tongue movements. Includes biting and pulling at the lure-presenter with teeth.
Time period	Interaction (oral)	Licking	2	Licking the object with visible tongue movements.
Time period	Interaction (body)	Face rubbing	3	Rubbing the cheeks, chin, neck and/or the whole rostrum on the object.
Time period	Interaction (body)	Holding	3	Keeping the object in a position in which it physically contacts the animal’s body for ≥ 3 s. Animal can use paw(s) to keep the object on the floor or elevated above the ground level while standing, sitting or lying.
Time period	Interaction (body)	Scratching	3	Scratching, striking or playing with the lure-presenter using their fore or hind paws.
Single event	Timepoint	End		First sound/sight of personnel as they return to cat pens to remove lure.

To identify whether cats responded differently to the five lure treatments, we fit generalised linear mixed models (GLMM) with a Tweedie distribution with a log link using the ‘glmmTMB’ package in R (Type III Wald chisquare tests) to each behaviour measure in separate analyses with lure treatment as predictors, and trial session (August or November) and individual cat ID as random factors (to account for the repeated measures on each animal). A sensitivity check was run with trial omitted, trial as a random factor, and trial as a fixed factor, but the effect of the lures was consistent between the three statistical tests (**Supplementary Table** S3). To control for differences in active times between cats, we repeated models investigating the total time spent in the zone and interacting with lures with active duration as an offset in the GLMMs. Cats that did not emerge, enter the zone or interact with lure-presenters were excluded from the latency models to avoid imposing artificial values (e.g. setting latency to the total trial duration (if cats didn’t emerge at all, their ‘latency’ was not calculable), but were included as zeros when modelling the duration of active trials, time spent in the zone, and time interacting with the lure-presenters. Estimated marginal means for lure type were obtained using the ‘emmeans’ package based on the fitted GLMMs and accounting for random effects of cat ID and trial session (Lenth et al. 2025). Pairwise comparisons were adjusted for multiple testing using Tukey’s method. We confirmed model fits using the ‘DHARMa’ package (Hartig and Lohse 2020).

### **Chemical Analysis of Lures**

GC-MS analyses were performed on an Agilent 8890 GC system coupled with a 5977 mass selective detector (MSD) (GC 8890/MSD 5977) equipped with an Agilent HP-5ms inert capillary column (30 m length, 0.25 mm internal diameter, 0.25 μm film, Agilent Technologies) for the identification and quantification of gas samples in both headspace and solvent injection analyses. Analysis specific parameters are detailed below in the respective methodologies for (a) Headspace solid phase microextraction (HS-SPME), and (b) Solvent extraction and analysis. For each of the four lure types, we completed three replicate GC-MS analysis for both the SPME and Solvent.

### Headspace Analysis - Headspace Solid Phase Microextraction (HS-SPME)

Headspace Solid Phase Microextraction (HS-SPME) method for gas phase sampling and analysis was adapted from Du et al. (2019). This was conducted using a 2 cm 50/30 µm divinylbenzene/carboxen/polydimethylsiloxane (DVB/CAR/PDMS) StableFlex™ fiber (Supelco, USA) housed in a 23-gauge needle. Prior to first use, the fibre was conditioned in the GC injector following the manufacturer’s recommendations to remove residual contaminants and ensure reproducible extraction performance. To control for background contamination and potential carryover effects, blank and carryover checks were routinely implemented by exposing the fibre to an empty 20 mL headspace vial throughout the analytical sequence.

For each lure, three 3.5 g samples each in 10 mL amber glass headspace vials were equilibrated at 35 °C within an air bath shaker for 5 min at 250 rpm. To assure the quality of the GC-MS data, diluted C7–C40 saturated alkane standards (100 µg/mL) were added in 10 µL quantities. To sample the VOCs the SPME fibre was exposed within the headspace of the sample vials for 1 h at 35 °C. Following the micro-extraction of the VOC analytes the SPME fibre was transferred directly to the GC inlet of the GC-MS, which was equipped with a split/splitless injector and a SPME inlet (Supelco), operated under splitless mode during the operation. The injection inlet was set at 160 °C and the GC purge valve was set to be switched on 0.5 min after injection. The GC oven held the initial temperature at 50 °C for 1 min, followed by an initial ramp to 100 °C at a rate of 5 °C/min, then a second ramp to 250 °C at a rate of 10 °C/min and then held at 250 °C for 5 min.

### Solvent Extraction and Analysis

Chemical analysis of the four commercial plant materials was based on Bol et al. (2017). Three 0.5 g replicates of each plant powder material were extracted with 3 mL of dichloromethane, by stirring at room temperature for 7 days within a sealed glass vial. The suspensions were then filtered through a syringe filter with 0.45 μm pore size and concentrated by evaporating the solvent at room temperature. The dry samples were reconstituted to 1.5 mL and injected to an Agilent 7890/5977 Gas Chromatography Mass Spectrometry (GC-MS) by an autosampler. Extract solvent solutions were analysed by GC-MS under the following operating conditions: He-flow: 1.2 ml/min, injector: 250 °C, transfer line 300 °C, electron energy 70 eV. The GC oven temperature was maintained at 50 °C for 5 min, then ramped to 320 °C at a rate of 5 °C/min and held at 320 °C for 2 min.

### GC-MS Data Analysis

**Fig. 2:**
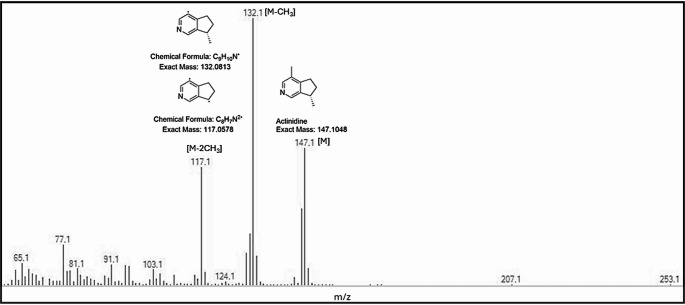
The extracted mass-spectrum of peak 21 in the solvent extracts are consistent with an expected mass-spectrum for Actinidine. The rational for this assignment is that actinidine has an exact mass of 147.1048 [M] ascribed to the signal at m/z 147.1. There appears to be fragment signals each at a loss of 15.0235, correlating to two demethylated fragments, 132.1 [M-CH_3_] and 117.1 [M-2CH_3_] respectively

GC-MS was performed by comparison of their mass spectra and retention indices (determined from a homologous series on n-alkanes; C7–C40) to commercial National Institute of Standards and Technology (NIST) 20 mass spectral libraries. Identifications were ascribed on the basis of similarity matches within the NIST data base. Actinidine is a previously reported compound within these plant species (Bol et al. 2017), yet is not present within the NIST database. As such, for the ascription of Peak 21 within the samples as actinidine, comparisons were made to literature examples of actinidine’s fragmentation pattern. The observations of signals corresponded to the calculated exact mass of the actinidine parent ion at 147.1 m/z, followed by two demethylated fragments, 132.1 [M-CH3] and 117.1 [M-2CH3] m/z respectively, consistent with that of literature reports for actinidine (Huth and Dettner 1990; Takatani et al. 2023) (see Fig. 2).

To assess differences in chemical composition of the plant products, we used Principal Components Analysis (PCA). Given that the two extraction methods (SPME and Solvent) produced different chemical profiles, separate PCAs were conducted for each method. For each PCA, a biplot was generated to visualise sample clustering along the first two principal components (PC1 and PC2), allowing interpretation of sample separation by plant product, and the contribution (loadings) of individual compounds to this multivariate space. To evaluate the influence of plant product type (lure treatment) on chemical composition, we performed permutational multivariate analysis of variance (PERMANOVA) separately for the SPME and solvent datasets. Due to limited sample sizes, post hoc pairwise comparisons between lure types were not conducted.

Values presented are means ± 1SE. Marginal mean estimates for each lure and their 95% confidence intervals are presented in **Supplementary Figure **S4 and pairwise contrasts in **Supplementary Table **S5.

## Results

### **Cat Behavioural Responses**

The cats were more active during the trials run in the afternoons (November trials); for 14 of the 50 trials in August, the cat did not emerge at all, compared with only 2 of the 50 trials in November. Trial session (August or November) and individual cat ID were accounted for as random factors in all the models. There was no statistical evidence of an effect of lure treatment on latency to emerge from shelter after personnel had left the experimental pen area (χ^*2*^_4_ = 9.03, *p* = 0.060), and no pairwise comparisons were significant. For those cats that approached the lure-presenter, there was no evidence of a lure treatment effect on latency to enter the lure zone (χ ^*2*^_4_ = 0.94, *p* = 0.919) or latency to interact with the lure (χ^*2*^_4_ = 1.21, *p* = 0.877). Further, there was no evidence of a lure effect on how long a cat spent outside its shelter (‘active duration’: χ^*2*^_4_ = 3.92, *p* = 0.417), which averaged 1.09 ± 0.65 (range 0–2.16) hours, although there were significant differences between individual cats (χ^*2*^_11_ = 53.09, *p* < 0.001).

There was a significant effect of lure treatment, however, on the amount of time spent in the lure zone (χ^*2*^_4_ = 13.45, *p* = 0.009), with cats spending longer in the zone with Silver vine-C (*est.* = 71.3 ± 43.9 s) compared to Silver vine-A (*est.* 19.1 ± 12.6 s, z = 3.25, *p* = 0.010), while the difference between Silver vine-C compared to the control did not reach statistical significance (*est.* = 26.1 ± 16.8 s, z = -2.58, *p* = 0.075). When considering time spent in the zone (including the offset: active duration), the differences between lure treatments did not reach statistical significance (χ^*2*^_4_ = 8.27, *p* = 0.082), although posthoc analysis suggested longer times associated with Silver vine-C compared to Silver vine-A (z = 2.74, *p* = 0.048).

**Fig. 3:**
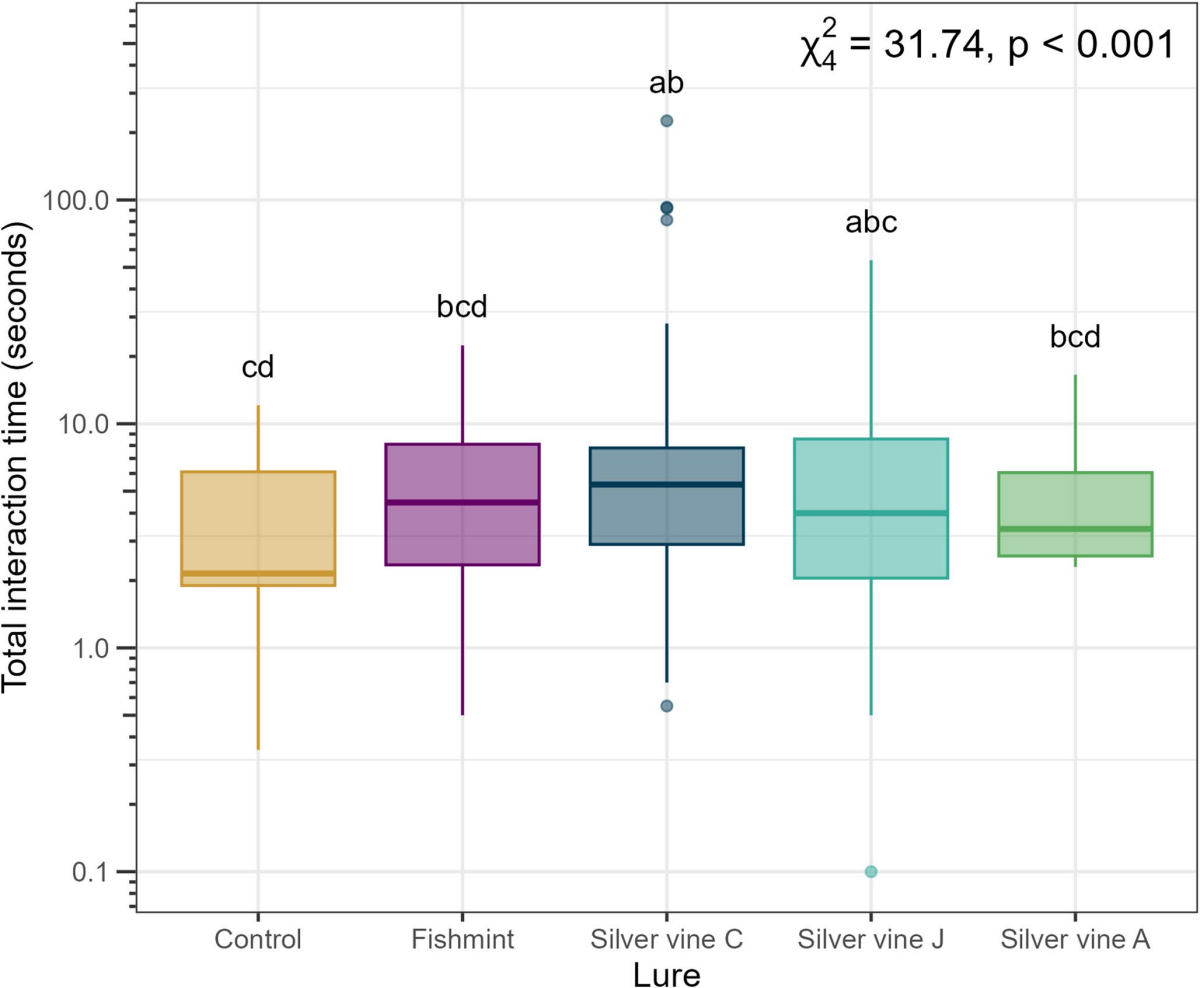
There was a significant effect of lure treatment on the amount of time spent interacting with each plant material. Lures are Fishmint (mix of five plant species), and three commercial silver vine (*Actinidia polygama*) products sourced from different regions — Silver vine-C (China), Silver vine-J (Japan), Silver vine-A (USA). Letters link treatments that were not significantly different from each other (Tukey’s post hoc analyses). There were 20 trials per lure (i.e., 2 trials per cat per lure, except where indicated in Table 2)

**Fig. 4:**
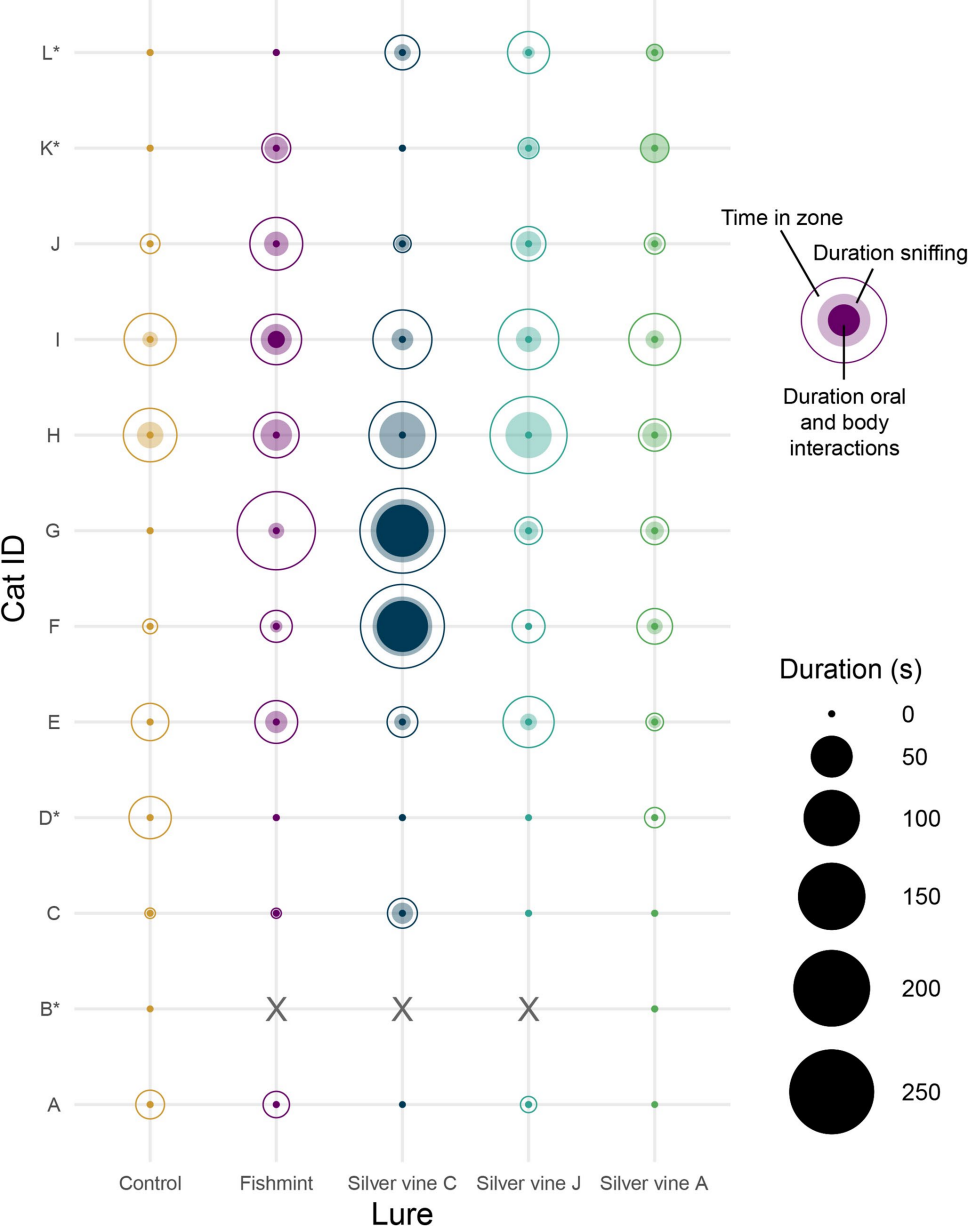
Representation of the total duration of time that an individual cat (*y* axis) spent within ~half a body length (open circles) and interacted (filled circles) with a control (nil treatment), Fishmint (mix of five plant species), and three commercial silver vine (*Actinidia polygama*) products sourced from different regions — Silver vine-C (China), Silver vine-J (Japan), Silver vine-A (USA) over each 2-hour duration trial. Largest circles: total amount of time within the zone (within ~half a body length of the lure-presenter); light-shaded circles: total amount of time sniffing; dark filled circles: total amount of time for oral and body ‘catnip responses’; smallest dots: cat emerged but did not approach to within half a body length of the lure. Values are averaged across the August and November trials (i.e., average value of 2 trials per cat per lure) except where indicated *Cat B (2 trials, X indicates trials that were not carried out) was replaced by cat K (8 trials, single presentation of control and Silver vine-A); cat D (5 trials, single presentation of each lure) was replaced by cat L (5 trials, single presentation of each lure)

**Fig. 5:**
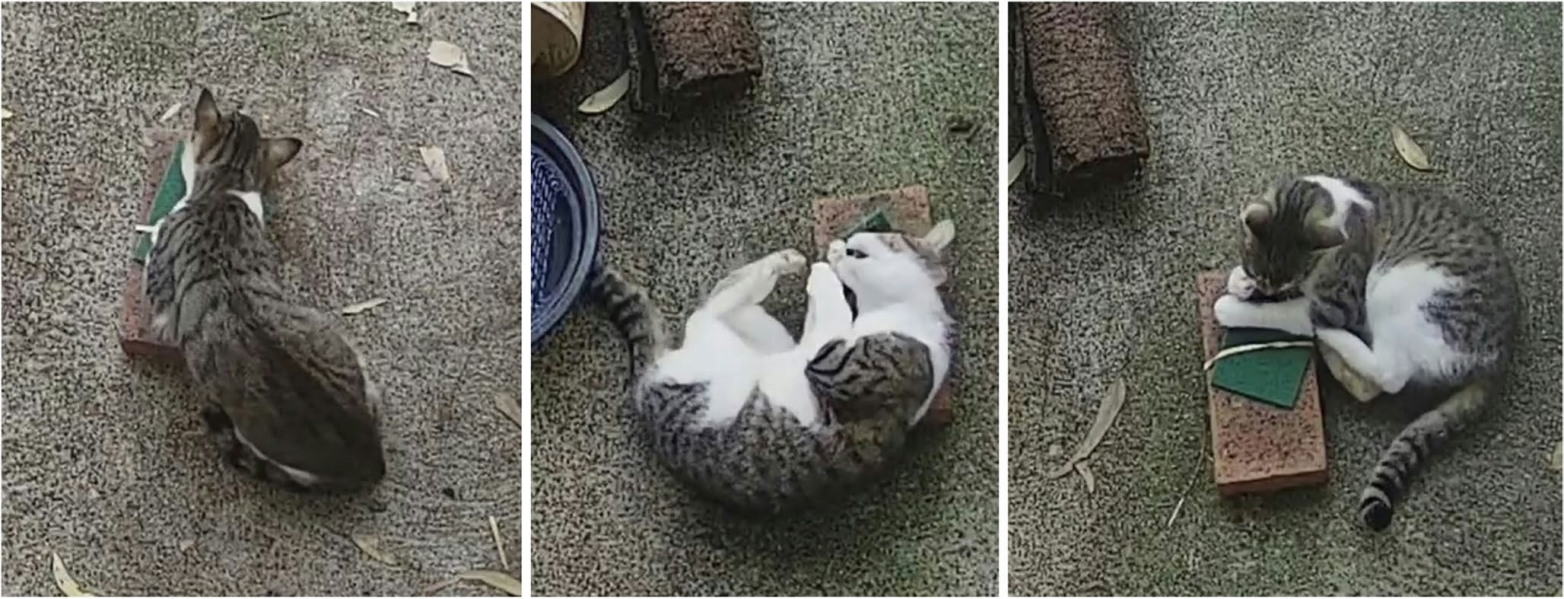
A feral cat (*Felis catus*) responding to presentation of silver vine (*Actinidia polygama*) powder

There was evidence for an effect of lure treatment on the total amount of time spent interacting with the lures expressed as a raw time value in seconds (χ^*2*^_4_ = 31.74, *p* < 0.001, Figs. 3 and 4) and when offset by active duration (χ^*2*^_4_ = 29.19, *p* < 0.001). Compared to the control (*est.*= 1.05 ± 0.97 s), interaction time was longer for Silver vine-J (*est.*= 9.08 ± 6.93 s; raw time: z = -3.08, *p* = 0.018; offset: z = -2.61, *p* = 0.068) and Silver vine-C (*est.*= 25.10 ± 18.40 s; raw time: z = -4.72, *p* < 0.001; offset: z = -4.48, *p* < 0.001), but not for Fishmint (*est.*= 7.09 ± 5.49 s; raw time: z = -2.67, *p* = 0.058; offset: z = -2.41, *p* = 0.112) or Silver vine-A (*est.*= 3.71 ± 2.98 s; raw time: z = -1.68, *p* = 0.445; offset: z = -1.54, *p* = 0.540). Pairwise analysis indicated longer interactions between Silver vine-C compared to Fishmint (raw time: z = -2.80, *p* = 0.041; offset: z = -2.75, *p* = 0.047) and Silver vine-A (raw time: z = 3.81, *p* = 0.001; offset: z = 3.62, *p* = 0.003), but not Silver vine-J (raw time: z = 2.37, *p* = 0.125; offset: z = 2.70, *p* = 0.053). No other significant pairwise differences between lure types were found. Interactions with the lure included sniffing, oral and body interactions (Figs. 4 and 5).

We recorded ‘positive responses’ (*sensu* Bol et al. 2017; who defined a positive response as sniffing, licking, biting, head shakes, or rubbing/rolling) in 18% of the control trials (2 of 11 cats that emerged), compared with 64% of the trials with Fishmint (7 of 11 cats that emerged), 80% of the trials with Silver vine-C (8 of 10 cats that emerged), 73% of the trials with Silver vine-A (8 of 11 cats that emerged), and 70% of the trials with Silver vine-J (7 of 10 cats that emerged). Cochran Q test indicated overall statistical significance of these responses (Q = 14.87, df = 4, p = 0.005), but McNemar pairwise post-hoc tests (with Bonferroni correction) indicated that only the difference between Silver vine-C and the Control reached statistical significance (*χ*^*2*^ = 8.10, p = 0.044).

### **Chemistry**

**Fig. 6:**
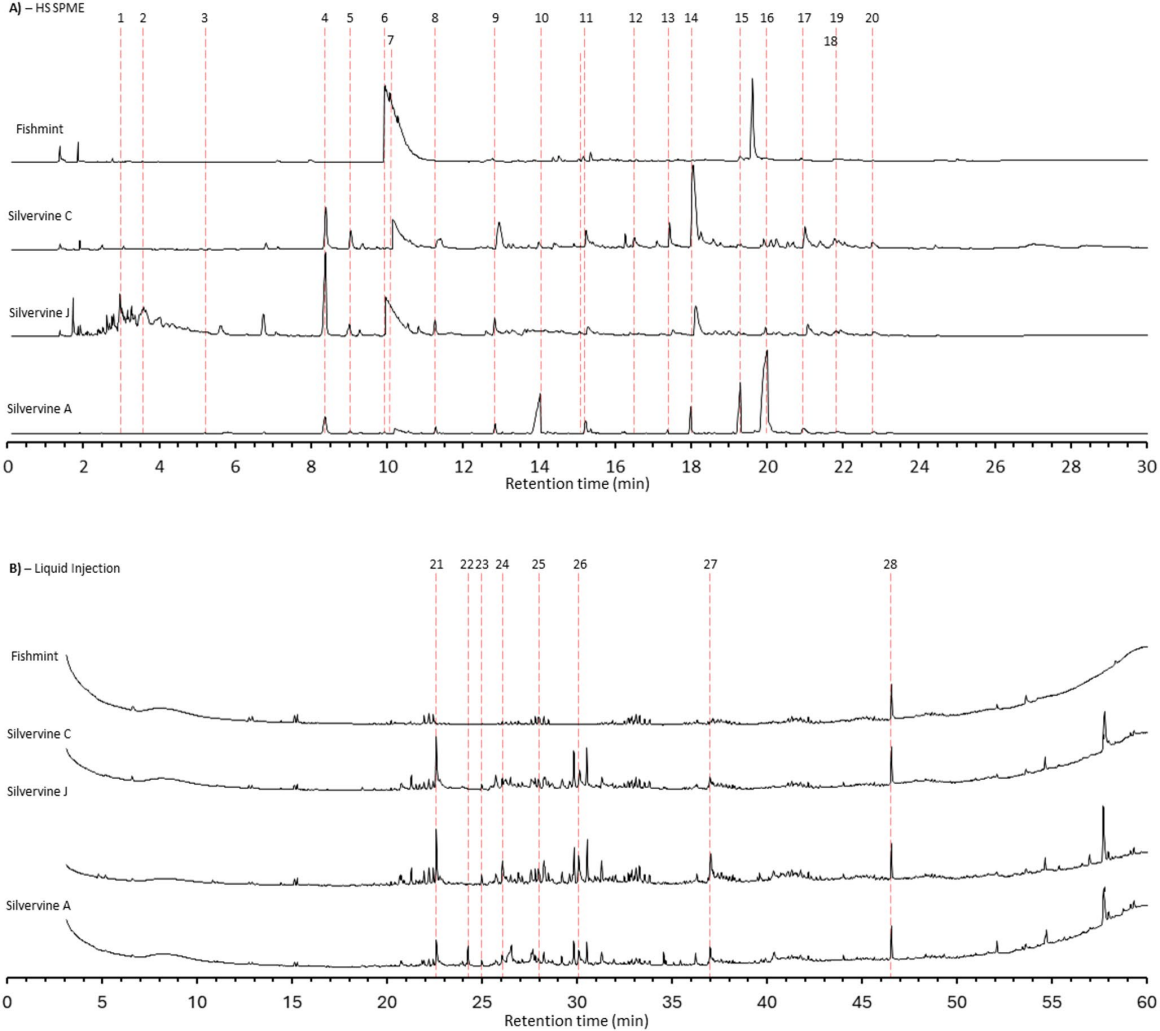
Typical GC profiles of Fishmint, and three commercial silver vine products sourced from different regions — Silver vine-C (China), Silver vine-J (Japan), Silver vine-A (USA) for (a) HS-SPME extract and (b) Solvent extract. Peak numbers correspond to compounds in Table 4. For each plot, the x-axis shows the retention time (minutes) and the y-axis the ionisation detector response. Not all peaks were found in each product; for instance, within chromatograph B (solvent extracts) peak 22 indicates nepetalactone for Silver vine-A

**Fig. 7:**
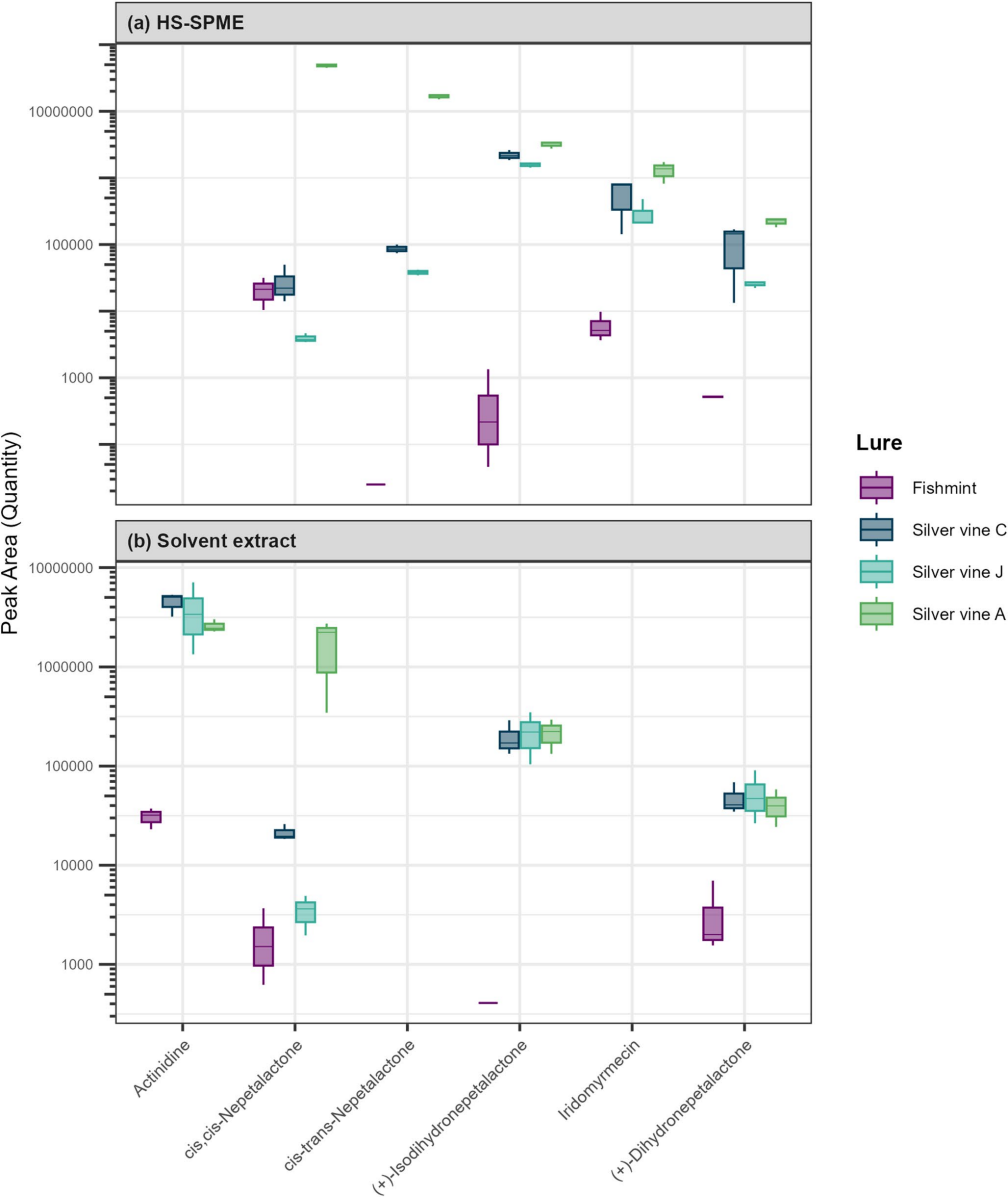
Relative quantity (peak area) for compounds that are believed to elicit behavioural responses in cats identified in four plant products by (**a**) headspace solid-phase microextraction (HS-SPME) and (**b**) solvent extract chemical analyses based on three samples per lure per analysis. Plotted are identified compounds with known or purported stimulating effect on cats (Bol et al. 2017; Lichman et al. 2020; Tucker and Tucker 1988)

Twenty compounds were identified by HS-SPME analysis and nine compounds by solvent extract (Table 4.; Fig. 6). Retention times and peak areas are reported in Supplementary Table S6. Six compounds recognised as bioactive in cats (Table 1) were detected from the samples, with five of these found in the HS-SPME extraction and four of these from the solvent extraction (Fig. 7). Four compounds — (+)-Isodihydronepetalactone, (+)-Dihydronepetalactone, cis-cis-Nepetalactone and 3-Oxabicyclo[5.3.0]decan-2-one, 9-methylene-, trans- — were detected using both HS-SPME and solvent extract. As the two extraction methods isolate compounds of differing physicochemical properties, the chemical profiles of HS-SPME and solvent extraction are described and analysed separately. Full PCA summary statistics are shown in Supplementary Table S7 and vector fitting results in Supplementary Table S8.

**Table 4.**
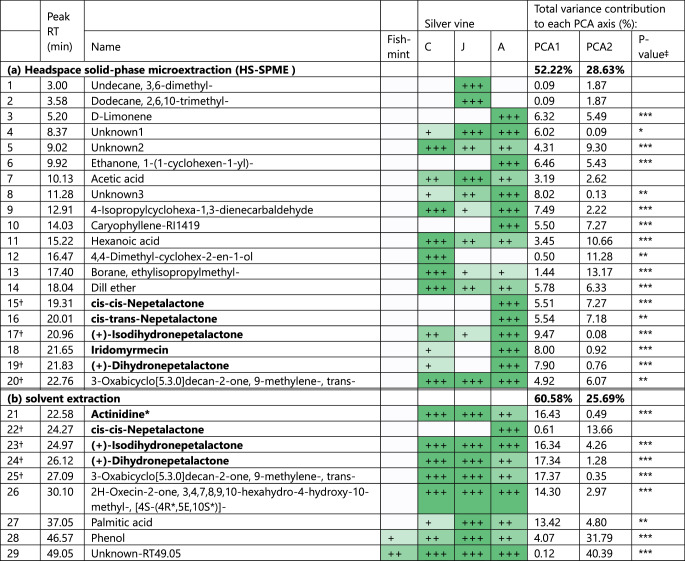
Contribution of chemical compounds to Principal Components Analyses (PCA) comparing four commercially available plant products, analysed using (a) headspace solid-phase microextraction (HS-SPME) and (b) Solvent extracts, using gas chromatography and mass spectrometry (GC-MS). Compounds with known or purported stimulating effect on cats are indicated in bold (Bol et al. 2017; Lichman et al. 2020; Tucker and Tucker 1988)

Semi-quantitative scoring: + low (bottom quartile), ++ medium (25-75% range) and +++ high (top quartile) concentrations, relative to other plant materials analysed in this study. RT retention time (in minutes)

‡ P-values indicate significance of the association of each compound with the overall ordination * *p*<0.05, ** *p*<0.01, *** *p*<0.001

†HS-SPME peaks 15, 17, 19, 20, and solvent extraction 22, 23, 24, 25 were the only compounds detected using both extraction methods

Green shading represents relative quantity of each compound across the four plant materials analysed (average of three samples); +++ top quartile (i.e. >0.75 of the maximum value recorded), ++ 0.5-0.75 of the maximum, + 0.5-0.25 of the maximum, blanks <0.25 of the maximum

* Peak 21 was manually assigned as actinidine; as this compound is not present within the NIST 2020 GC-MS database, but was anticipated within the sample based on literature reports

### HS-SPME Extraction

The first two principal component analysis axes for the HS-SPME extractions collectively explained 80.85% of the variance (PC1: 52.22% and PC2: 28.63%). Seventeen of the 20 chemicals were correlated with the PCA ordination (Table 4.a), with three bioactive compounds strongly correlated with variation captured by the ordination (*r*^2^ > 0.99): Peak 17 ((+)-Isodihydronepetalactone, p = 0.001), Peak 15 (cis, cis-Nepetalactone, p = 0.002) and Peak 16 (cis-trans-Nepetalactone, p = 0.002).

**Fig. 8:**
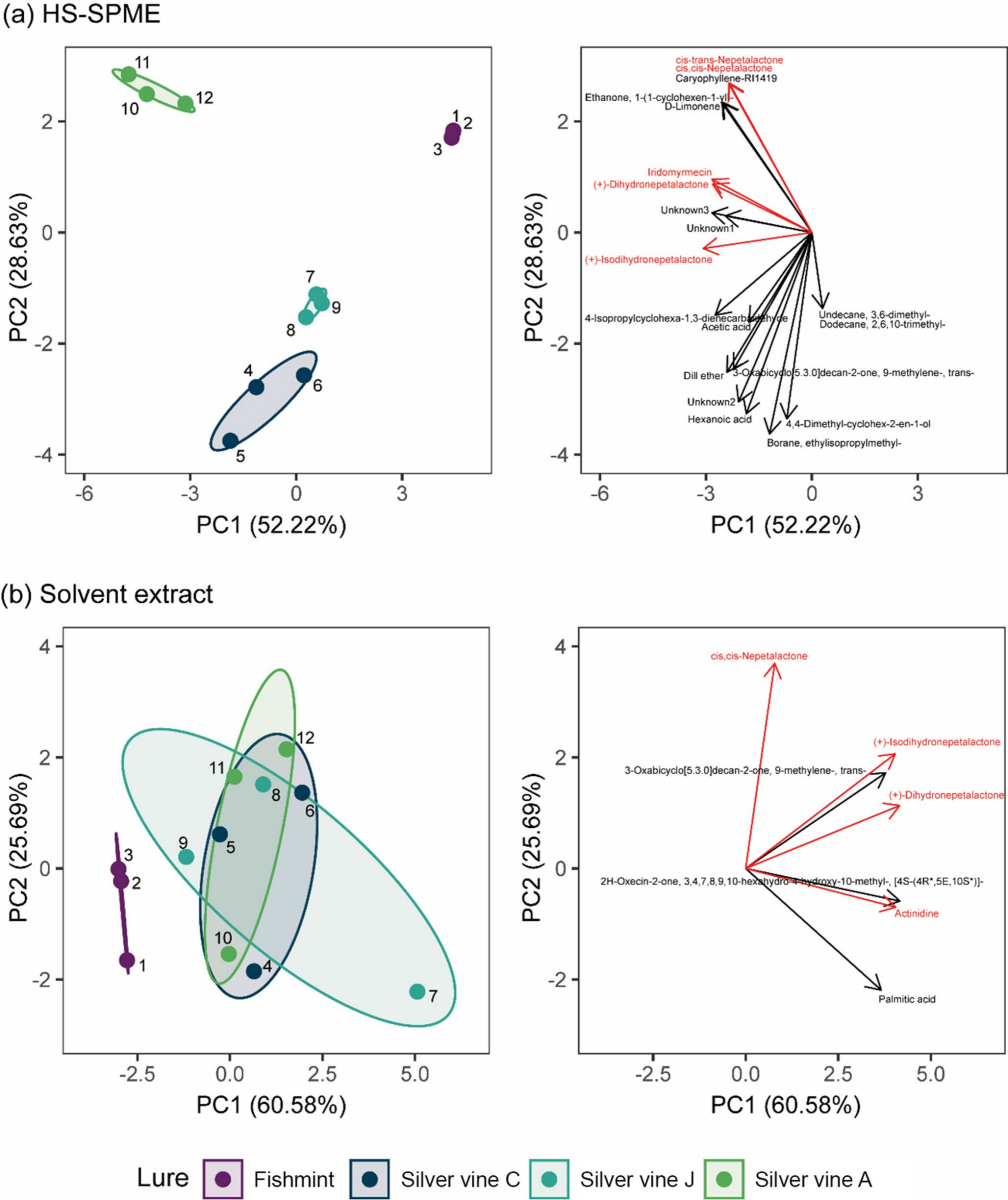
Separation of the odorous control (Fishmint) from the three silver vine products on the first two Principal Components factors for (a) headspace solid-phase microextraction (HS-SPME) and (b) solvent extraction. Ellipses show 95% confidence range for each lure group’s mean. The three commercial silver vine products were sourced from different regions — Silver vine-C (China), Silver vine-J (Japan), Silver vine-A (USA). Numbers show the sample number (three replicates of each plant material. Arrows show the peak loadings for PC1 (*x*-axis) and PC2 (*y*-axis), with compounds that are believed to elicit behavioural responses in cats (Bol et al. 2017; Lichman et al. 2020; Tucker and Tucker^1988^) shown in red. Note: there are many correlated compounds showing overlapping arrows

There was a significant effect of lure type on the chemical composition of HS-SPME extract (*F*_3,8_ = 36.93, *p* < 0.001) with all four plant materials showing distinct separation in the PCA ordination (Fig. 8a). Silver vine-A was correlated with negative values of PC1 and positive values of PC2 and had relatively high levels of Peak 15 (cis-cis-Nepetalactone), Peak 16 (cis-trans-Nepetalactone) and Peak 17 ((+)-Isodihydronepetalactone) (Figs. 7a and 8a), which is consistent with this product being a blend of catnip and silver vine. In contrast, the other three lures were positively loaded on PC1, and had relatively low amounts of Peak 15 and Peak 16.

### Solvent Extraction

The first two principal component analysis axes for the solvent extractions collectively explained 86.26% of the variance (PC1: 60.58% and PC2: 25.69%). Eight out of the nine chemicals had strong correlations with the ordination (Table 4.b). Three bioactive compounds were correlated with the ordination (*r*^2^ > 0.90): Peak 23 ((+)-Isodihydronepetalactone, *r*^2^ = 0.99, p = 0.001), Peak 24 ((+)-Dihydronepetalactone, *r*^2^ = 0.98, *p* = 0.001), and Peak 21 (Actinidine, *r*^2^ = 0.91, *p* = 0.001). The last bioactive compound Peak 22 (cis, cis-Nepetalactone) was not strongly correlated with the ordination (*r*^2^ = 0.35, *p* = 0.184).

There was a significant effect of lure type on the chemical composition of the solvent extract (*F*_3,8_ = 2.45, *p* = 0.037). Fishmint had low quantities of all bioactive compounds (Fig. 7b) and was distinctly separate from the three silver vine lures in the ordination, while the three silver vine lures exhibited overlapping characteristics (Fig. 8b).

## Discussion

The euphoric ‘catnip’ response in the domestic cat, linked to volatile compounds produced by several plants (Bol et al. 2017; Lichman et al. 2020), could provide a valuable management tool for feral cats, with an effective lure potentially improving activity around camera traps, increasing detections and therefore robustness of monitoring methods. In this study, we compared the behavioural responses of feral cats to three commercially available dried silver vine products, an odorous control (Fishmint), and a no plant material treatment. There was some evidence that the plant materials increased the amount of time cats spent in proximity to and interacting with the lure (within the ‘lure zone’), especially with silver vine-C compared to the control (no plant material). Responses towards the three silver vine products suggest differences in the chemistry of these plant materials had an influence on cat behaviour. Chemical profiles for the four plant products revealed six compounds recognised as bioactive in cats across headspace and solvent extractions of each of the three silver vine products.

Three nepetalactone isomers were detected in commercial dried silver vine products, including cis-cis-nepetalactone, cis-trans-nepetalactone, (+)-isodihydronepetalactone, and (+)-dihydronepetalactone. We recovered cis-trans-nepetalactone only in the headspace analysis, while the other two compounds were identified in both headspace and solvent extracts. Nepetalactone isomers have been recovered from catnip and silver vine previously (e.g., Keesey et al. 2019) and have been associated with the catnip response in felids (reviewed by Tucker and Tucker 1988; Uenoyama et al. 2021). Nepetalactol is a common biosynthetic precursor of iridoid monoterpenes and shares structural similarities with cis-trans nepetalactone (Lichman et al. 2020). Fresh silver vine has been reported to contain high levels of nepetalactol (Keesey et al. 2019; Uenoyama et al. 2021), but during drying or prolonged storage, this compound may oxidise to nepetalactone (Hernández Lozada et al. 2022). The nepetalactone found in our study, from commercial dried silver vine products, is therefore likely a result of exposure to thermal processing during drying or storage. This highlights the importance of controlling for the condition and processing history of plant material, as it can significantly affect the chemical composition of plant-based lures, and potentially impact their biological effectiveness and shelf life.

Actinidine was also tentatively identified in our commercial products (Peak 21). Silver vine-C and one sample of Silver vine-J (sample 10) had relatively higher amounts of actinidine than the other plant materials, and cats were found to spend more time interacting with Silver vine-C, anecdotally suggesting actinidine may contribute to lure attractiveness. Actinidine is previously reported within these plant species (Bol et al. 2017), but is not present within the NIST 2020 GC-MS database. As such, ascription of Peak 21 to actinidine was performed manually on the basis of the mass fragmentation pattern of actinidine, supported both by calculated values for the molecular ion (actinidine parent ion [M] = 147.1 Da), and two demethylated fragments at 132.1 ([M-CH_3_]) and 117.1 ([M-2CH_3_]; and by comparison to literature evidence: Huth and Dettner 1990; Takatani et al. 2023) (Fig. 2). While actinidine has been identified in multiple bioactive plant materials (Table 1), the presence of actinidine in our commercial products could be an artifact of how they were processed. Shi et al. (2020) demonstrated that actinidine can be formed from iridodial precursors, particularly nepetalactone in *A. polygama* and *N. cataria*, handled under thermal conditions exceeding 100 °C. We are uncertain of the thermal processing history of the commercial silver vine plant products analysed in this study; however, thermal processing in the production of the products could account for the generation of actinidine as detected.

Another important compound found in our commercial products was iridomyrmecin. This compound was detected in the headspace of all silver vine samples, with the highest concentrations found in Silver vine-A, a product containing both silver vine and catnip. Iridomyrmecin is not known from catnip (Bol et al. 2017; Keesey et al. 2019; Uenoyama et al. 2021), but has been consistently reported in silver vine (Bol et al. 2017; Keesey et al. 2019; Uenoyama et al. 2021). Beyond silver vine, iridomyrmecin and its isomers have been found in other plants known to elicit cat-attracting behaviour, including Valerian root *Valeriana officinalis* (Bol et al. 2017) and Indian nettle *Acalypha indica* (Scaffidi et al. 2016). Presence of iridomyrmecin and its isomers across multiple cat-attracting plants suggests a role for these compounds in mediating behavioural responses in felines.

There are many factors that can influence which compounds are detected in a plant product. As outlined above for nepetalactone and actinidine, how the product is handled during processing can change its chemical signature. In addition to the effects of sample processing, the extraction method itself can influence which compounds are detected, as it introduces a selective bias toward compounds with specific physicochemical properties (e.g., Jennings and Filsoof 1977; Omar et al. 2016). Different components will be extracted with varying efficiency according to the solvent used, with volatile oil components more likely extracted with a polar alcohol solvent, while aqueous extracts will result in larger amounts of amino acids isolated (Houghton 1999). For instance, HS-SPME is a sorptive technique that samples volatile compounds in the gas phase, with selectivity influenced by volatility, polarity (e.g., Tir et al. 2012), and affinity to the fibre. In contrast, liquid extraction of plant powders using dichloromethane enables recovery of less volatile compounds but is less effective for highly polar compounds or insoluble salts. As such, the relative abundance of compounds may be influenced, not only by their presence in the sample, but also by the extraction technique used. Furthermore, different compounds ionise with varying efficiency in the mass spectrometer, which can also affect their detectability. In our study, the distribution of compounds differed between the two extraction methods used, highlighting the need for standardised extraction protocols when comparing chemical compositions across studies.

In addition to processing and analysis, the plant itself may show significant variation in chemical composition (Houghton 1999). Previous publications report marked differences in chemical profile between studies testing the same species of plants (Table 1). Part of this variation may be due to plant parts (e.g., leaf, fruit, roots) naturally containing more or less of the bioactive compounds (we note that many commercial products do not identify which plant part they are derived from). For example, infestation of fruit by the matatabi fruit gall midge, *Pseudasphondylia matatabi* produces new chemical compounds in the plant (Abramson et al. 2012). Comparing different plant materials, Bol et al. (2017) noted marked responses to a dried powder made from parasitic insect-galled silver vine fruit, but minimal responses to other plant parts (e.g., leaves) for nine cats. By contrast, Uenoyama et al. (2021) showed strong cat responses to synthesised nepetalactol, which they also extracted from silver vine leaves, showing that silver vine leaf material can include bioactive compounds. Perhaps leaf age may influence differences in attractiveness to cats. Seasonal changes in plant composition could also influence chemical composition. Harvest time influenced the accumulation of secondary metabolites (nepetalic acid, nepetalactone, and dihydronepetalactone) differentially for *Nepeta cataria* genotypes (Patel et al. 2022). Other plant composition changes with season (e.g., sugar and starch concentrations in *Actinidia* spp., Boldingh et al. 2000), suggesting that time of year effects could also be observed for other cat attractive plants.

In testing the bioactivity of these plant compounds, it is worth recognising that marked differences in behavioural responses between cat populations have been identified. There is a genetic link with receptiveness to nepetalactone (believed to be the active ingredient of *Nepeta cataria*) and the ‘catnip response’ is pharmacologically initiated via µ-opioid receptors (Uenoyama et al. 2021). This may be the reason why *Nepeta cataria* is only effective on one-third to two-thirds of cats (Bol et al. 2017; Wickramaratne et al. 2022). Similarly, Rupasinghe et al. (2022) also quantified differences in allele frequencies for OR10K1, OR10V1, and OR2B11 olfactory receptor genes for cats categorised as ‘responders’ or ‘non-responders’ to *Acalypha indica*.

For cats that did emerge from their shelter in our study, there was no significant effect of the lure type on latency to enter the lure zone, suggesting that the lures were likely to only be effective over a short distance. This limited attraction radius is a critical consideration when designing lures for field deployment, where environmental factors such as wind and temperature could influence dispersal of the lure. Lure effectiveness involves trade-offs between highly volatile compounds and those that would act over shorter ranges but persist longer. For example, increased licking, chewing, and face rubbing was observed in two thirds of cats exposed to a toy embedded with freshly prepared *Acalypha indica* roots (Rupasinghe et al. 2022) or gauze with an extract of the roots (Wickramaratne et al. 2022). However, Scaffidi et al. (2016) reported that the chemical composition of *Acalypha indica* changed over a short period of time due to the high proportion of volatile chemicals, and therefore cat responses would likely change as the lure aged. Compared with high volatility compounds, relatively low volatility compounds, such as actinidine (which has a high boiling point), may be more suitable for field-based lures due to greater environmental persistence.

Storage and longevity of plant-based lures are also key concerns, as iridoid compounds like nepetalactone are susceptible to photodegradation, hydrolysis, and oxidative breakdown (Lockhart et al. 2024). Nepetalactone is also prone to hydrolysis and oxidative degradation in liquid formulations, leading to the formation of less active hydroxy acids and other metabolites (Patience et al. 2018). This makes using a liquid-based lure matrix more challenging, and consideration will need to be given to solvent selection and the potential inclusion of antioxidants or encapsulating agents to maintain compound integrity of the bioactive ingredients. Although powdered plant material likely offers improved chemical stability, powdered lures present practical challenges for field deployment. Optimising lure matrix formulation approaches that balance bioactive compound stability with ease of lure deployment will be important for advancing this lure in terms of long-term field deployment.

If requiring baits/lures for large-scale deployment, this raises the question of whether there is efficiency in relying on natural extracts – representing a mixture of bioactive compounds in varying concentrations – or pure compounds (artificially synthesised) that can be regulated. It is possible that a single component is attractive to cats (as shown by Uenoyama et al. 2021), but the cocktail effect of the plant compounds, especially given the similar chemistry of the iridoids (7-methylcyclopentapyranones, including nepetalactone and iridomyrmecin isomers) and monoterpenoid alkaloids (7-methyl-2-pyrindines; including actinidine) may provide a stronger effect. The answer to this question is therefore likely to rely on costs and demonstrated efficacy of particular plant strains and processing methods.

Finally, camera traps and control tools would work best with more time to respond to cat presence. Therefore the duration of interaction will influence feasibility of management options. All the lures investigated in this study initiated at least one interaction of more than 7 s in duration (43 of 220 interactions were > 7 s). Further investigation of the lures will likely be required in this regard.

One limitation of our experimental design was the change in trial timing and recording duration between sessions. This adjustment was made deliberately to leverage increased cat activity later in the day during the November trials, but the inconsistency in timing (shorter recording durations but more active cats) may have influenced behavioural response data, such as records of motivation to explore or interact with lures. Although we accounted for trial session as a random effect in our models and found that lure treatment effects were consistent across sessions, caution is needed when generalising findings to field conditions.

Overall, silver vine products elicited small but consistent behavioural responses from feral cats, demonstrating their potential as olfactory lures for invasive species management. Our chemical analyses revealed variation in bioactive iridoid compounds among commercial silver vine products, likely contributing to differences in cat responsiveness. These findings underscore the importance of product composition and processing in determining lure efficacy, and support the integration of silver vine-based lures into detection and monitoring programs for feral cats. Future research should evaluate lure performance under field conditions and explore strategies to optimise the chemical stability and attractiveness of silver vine to feral cats.

## Supplementary Material

Below is the link to the electronic supplementary material.

Supplementary File 1 (DOCX 873 KB)

## Data Availability

Data is provided within the manuscript or supplementary information files.
