# Supplementary material for: Feral Cat Responses to Silver Vine: Implications for Feral Cat Management
Source: J Chem Ecol. 2026 Apr 13;52(2):34. doi: 10.1007/s10886-026-01707-5 (PMC13076403; doi:10.1007/s10886-026-01707-5)
Supplement: Supplementary file 1 — (DOCX 873 KB) [file 10886_2026_1707_MOESM1_ESM.docx]

# Supplementary material

**
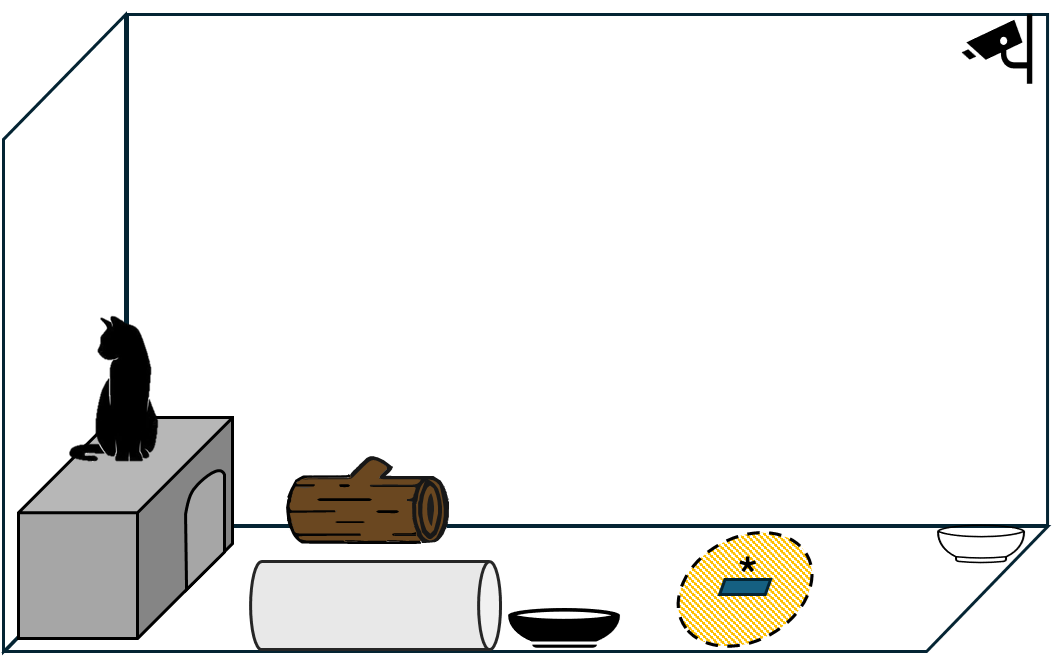
**

**Supplementary Figure S1** Schematic of cat pens 1.0 x 3.0 x 2.3 m (W x L x H) used in the trials. Each pen contained a kennel for shelter, enrichment including a PVC pipe for hiding and a wooden branch for scratching and climbing, water provided ad libitum (black bowl), and daily food (white bowl). The scourer pad (*‘lure-presenter’) was placed approximately 2m away from the kennel shelter. The yellow hashed circle denotes the area considered to be within the ‘interaction zone’. Each pen had a dedicated CCTV camera to monitor cat behaviour.

**Supplementary Table S2** Lures were presented to cats over two five-day periods per trial session. Lures were rotated in alternating pens in a consistent order: Silver vine-A (USA), Silver vine-C (China), Silver vine-J (Japan), ‘Fishmint’ powder and a control where no lure was presented on the scourer.

|  | **Day** | **Pen 1** | **Pen 2** | **Pen 3** | **Pen 4** | **Pen 5** | **Pen 6** | **Pen 7** | **Pen 8** | **Pen 9** | **Pen 10** |
| --- | --- | --- | --- | --- | --- | --- | --- | --- | --- | --- | --- |
| **Week 1** | **1** | A |  | C |  | J |  | Fishmint |  | Control |  |
|  | **2** |  | A |  | C |  | J |  | Fishmint |  | Control |
|  | **3** | Control |  | A |  | C |  | J |  | Fishmint |  |
|  | **4** |  | Control |  | A |  | C |  | J |  | Fishmint |
|  | **5** | Fishmint |  | Control |  | A |  | C |  | J |  |
|  | |  |  |  |  |  |  |  |  |  |  |
| **Week 2** | **1** |  | Fishmint |  | Control |  | A |  | C |  | J |
|  | **2** | J |  | Fishmint |  | Control |  | A |  | C |  |
|  | **3** |  | J |  | Fishmint |  | Control |  | A |  | C |
|  | **4** | C |  | J |  | Fishmint |  | Control |  | A |  |
|  | **5** |  | C |  | J |  | Fishmint |  | Control |  | A |

**Supplementary Table S3** We performed a sensitivity check for trial sessions. The main effects for Lure and Trial reported here for model sets with (a) trial omitted from the model, (b) trial treated as a random factor and (c) trial treated as a fixed factor. All models show consistent effects of lure. Model set (b) was retained in our analysis. Significant effects are shown in bold.

| **Model** | **Predictor** | **(a) Trial omitted** | | **(b) Trial as random factor** | | **(c) Trial as fixed factor** |
| --- | --- | --- | --- | --- | --- | --- |
| Latency to emerge | Lure | χ*^2^*_4_ = 1.21, p = 0.876 | | χ*^2^*_4_ = 9.03, p = 0.060 | | χ*^2^*_4_ = 9.48, p = 0.050 |
|  | Trial | *NA* | | *NA* | | **χ*^2^*_1_ = 72.20, p < 0.001** |
|  |  |  | |  | |  |
| Latency to enter zone | Lure | χ*^2^*_4_ = 0.94, p = 0.919 | | χ*^2^*_4_ = 0.94, p = 0.919 | | χ*^2^*_4_ = 0.97, p = 0.914 |
|  | Trial | *NA* | | *NA* | | χ*^2^*_1_ = 0.08, p = 0.782 |
|  |  |  | |  | |  |
| Latency to interact | Lure | χ*^2^*_4_ = 1.93, p = 0.749 | | χ*^2^*_4_ = 1.21, p = 0.877 | | χ*^2^*_4_ = 1.49, p = 0.828 |
|  | Trial | *NA* | | *NA* | | χ*^2^*_1_ = 0.11, p = 0.735 |
|  |  |  | |  | |  |
| Active duration | Lure | χ*^2^*_4_ = 3.90, p = 0.420 | | χ*^2^*_4_ = 3.92, p = 0.417 | | χ*^2^*_4_ = 3.99, p = 0.408 |
|  | Trial | *NA* | | *NA* | | χ*^2^*_1_ = 1.70, p = 0.193 |
|  |  |  | |  | |  |
| Zone Duration | Lure | **χ*^2^*_4_ = 12.33, p = 0.015** | | **χ*^2^*_4_ = 13.45, p = 0.009** | | **χ*^2^*_4_ = 13.50, p = 0.009** |
|  | Trial | *NA* | | *NA* | | **χ*^2^*_1_ = 22.67, p < 0.001** |
|  |  |  | |  | |  |
| Zone duration (offset) | Lure | χ*^2^*_4_ = 8.06, p = 0.089 | | χ*^2^*_4_ = 8.27, p = 0.082 | | χ*^2^*_4_ = 8.26, p = 0.082 |
|  | Trial | *NA* | | *NA* | | **χ*^2^*_1_ = 24.71, p < 0.001** |
|  |  |  | |  | |  |
| Interaction (duration) | Lure | **χ*^2^*_4_ = 29.31, p < 0.001** | | **χ*^2^*_4_ = 31.74, p < 0.001** | | **χ*^2^*_4_ = 31.96, p < 0.001** |
|  | Trial | *NA* | | *NA* | | **χ*^2^*_1_ = 13.88, p < 0.001** |
|  |  |  | |  | |  |
| Interaction duration (offset) | Lure | **χ*^2^*_4_ = 25.88, p < 0.001** | | **χ*^2^*_4_ = 29.19, p < 0.001** | | **χ*^2^*_4_ = 29.45, p < 0.001** |
|  | Trial | *NA* | | *NA* | | **χ*^2^*_1_ = 17.81, p < 0.001** |
|  |  |  |  | |  | |


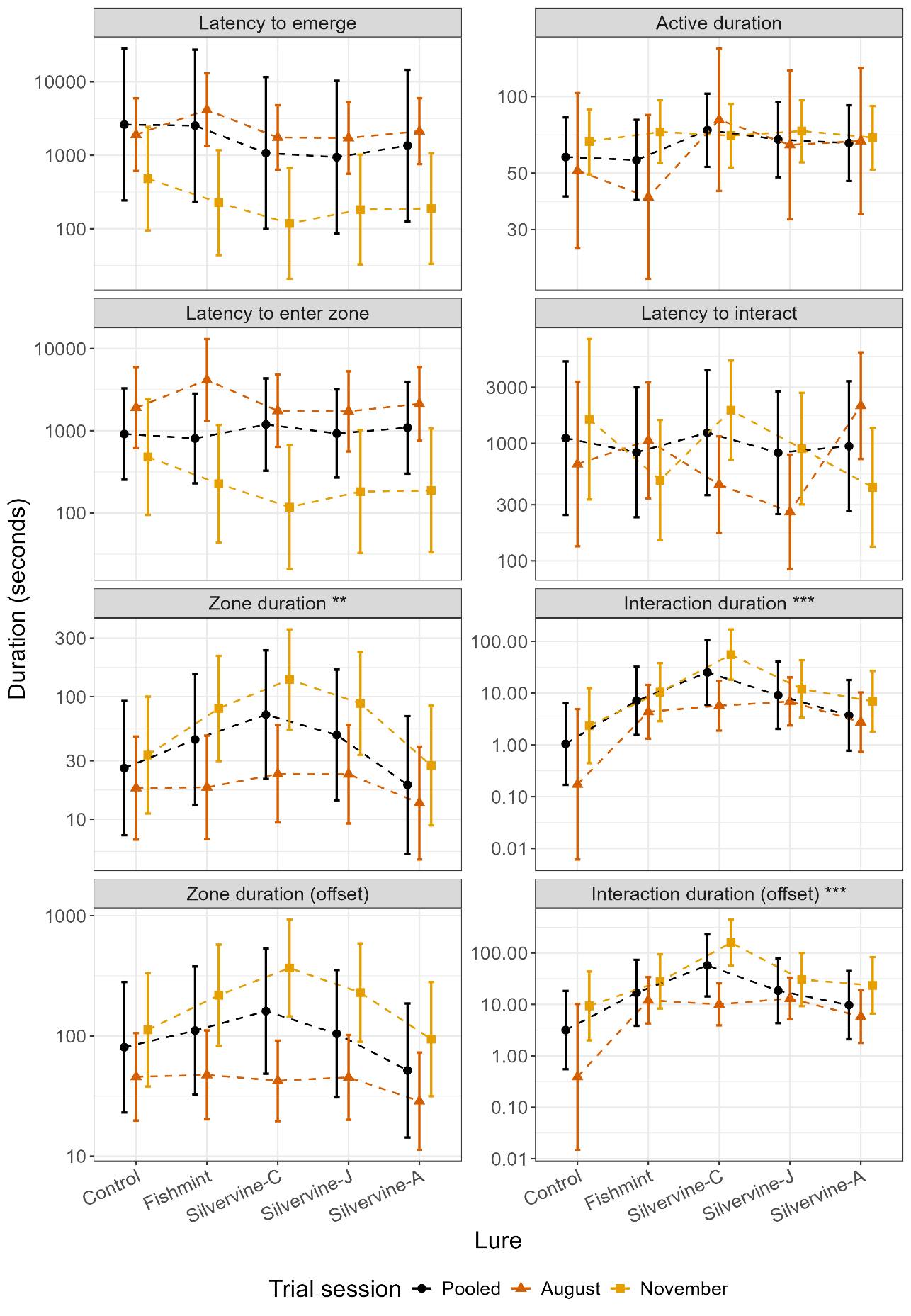


**Supplementary Figure S4** Estimates for each lure derived from the overall pooled model, compared to August and November trial sessions. Dot and whiskers show the estimated marginal means and their 95% confidence intervals. Dashed lines show general pattern between lure types. The y-axis is on the log-scale. Asterisks show models with a significant effect of lure where * p<0.05, ** p<0.01, *** p<0.001.

**Supplementary Table S5** Pairwise contrasts among lures for each behavioural model, shown for the overall pooled analysis and for each trial session (August and November). Ratios on the response scale, standard error (SE), 95% confidence interval bounds (CI), z-ratio and adjusted Tukey p-values are reported for each pairwise comparison. Significant pairwise contrasts are shown in bold.

| **Model** | **Trial** | **Pairwise Contrast** | **Ratio** | **SE** | **Lower CI** | **Upper CI** | **Z-ratio** | **p-value** |
| --- | --- | --- | --- | --- | --- | --- | --- | --- |
| Latency to emerge | Pooled | Control / Fishmint | 3.60 | 1.55 | 1.11 | 11.64 | 0.07 | 1.000 |
| Latency to emerge | Pooled | Control / Silver vine-C | 8.51 | 3.65 | 2.65 | 27.38 | 2.08 | 0.227 |
| Latency to emerge | Pooled | Control / Silver vine-J | 9.69 | 4.39 | 2.82 | 33.35 | 2.26 | 0.159 |
| Latency to emerge | Pooled | Control / Silver vine-A | 6.72 | 2.90 | 2.08 | 21.76 | 1.53 | 0.546 |
| Latency to emerge | Pooled | Fishmint / Silver vine-C | 8.25 | 3.65 | 2.46 | 27.59 | 1.94 | 0.294 |
| Latency to emerge | Pooled | Fishmint / Silver vine-J | 9.39 | 4.37 | 2.63 | 33.47 | 2.13 | 0.209 |
| Latency to emerge | Pooled | Fishmint / Silver vine-A | 6.51 | 2.80 | 2.02 | 21.02 | 1.46 | 0.592 |
| Latency to emerge | Pooled | Silver vine-C / Silver vine-J | 3.97 | 1.80 | 1.15 | 13.69 | 0.29 | 0.999 |
| Latency to emerge | Pooled | Silver vine-C / Silver vine-A | 2.75 | 1.18 | 0.86 | 8.87 | -0.55 | 0.982 |
| Latency to emerge | Pooled | Silver vine-J / Silver vine-A | 2.42 | 1.11 | 0.69 | 8.46 | -0.80 | 0.932 |
| Latency to emerge | August | Control / Fishmint | 0.85 | 0.45 | 0.20 | 3.63 | -1.46 | 0.586 |
| Latency to emerge | August | Control / Silver vine-C | 2.03 | 1.02 | 0.51 | 8.02 | 0.18 | 1.000 |
| Latency to emerge | August | Control / Silver vine-J | 2.06 | 1.22 | 0.41 | 10.42 | 0.18 | 1.000 |
| Latency to emerge | August | Control / Silver vine-A | 1.67 | 0.85 | 0.42 | 6.65 | -0.21 | 1.000 |
| Latency to emerge | August | Fishmint / Silver vine-C | 4.41 | 2.30 | 1.07 | 18.25 | 1.66 | 0.457 |
| Latency to emerge | August | Fishmint / Silver vine-J | 4.48 | 2.68 | 0.87 | 22.96 | 1.47 | 0.581 |
| Latency to emerge | August | Fishmint / Silver vine-A | 3.62 | 1.86 | 0.89 | 14.68 | 1.31 | 0.688 |
| Latency to emerge | August | Silver vine-C / Silver vine-J | 1.89 | 0.93 | 0.49 | 7.18 | 0.03 | 1.000 |
| Latency to emerge | August | Silver vine-C / Silver vine-A | 1.52 | 0.70 | 0.44 | 5.33 | -0.43 | 0.993 |
| Latency to emerge | August | Silver vine-J / Silver vine-A | 1.50 | 0.78 | 0.36 | 6.19 | -0.41 | 0.994 |
| Latency to emerge | November | Control / Fishmint | 5.99 | 4.23 | 0.87 | 41.10 | 1.06 | 0.826 |
| Latency to emerge | November | Control / Silver vine-C | 11.46 | 9.01 | 1.34 | 97.90 | 1.78 | 0.385 |
| Latency to emerge | November | Control / Silver vine-J | 7.44 | 5.81 | 0.88 | 62.70 | 1.24 | 0.729 |
| Latency to emerge | November | Control / Silver vine-A | 7.22 | 5.82 | 0.80 | 65.00 | 1.16 | 0.772 |
| Latency to emerge | November | Fishmint / Silver vine-C | 5.41 | 4.33 | 0.61 | 48.00 | 0.81 | 0.927 |
| Latency to emerge | November | Fishmint / Silver vine-J | 3.51 | 2.81 | 0.40 | 31.10 | 0.27 | 0.999 |
| Latency to emerge | November | Fishmint / Silver vine-A | 3.41 | 2.85 | 0.35 | 33.50 | 0.22 | 0.999 |
| Latency to emerge | November | Silver vine-C / Silver vine-J | 1.83 | 1.62 | 0.17 | 20.30 | -0.49 | 0.988 |
| Latency to emerge | November | Silver vine-C / Silver vine-A | 1.78 | 1.57 | 0.16 | 19.70 | -0.53 | 0.985 |
| Latency to emerge | November | Silver vine-J / Silver vine-A | 2.74 | 2.47 | 0.24 | 31.90 | -0.03 | 1.000 |
| Latency to enter zone | Pooled | Control / Fishmint | 1.23 | 0.60 | 0.33 | 4.66 | 0.25 | 0.999 |
| Latency to enter zone | Pooled | Control / Silver vine-C | 0.84 | 0.39 | 0.24 | 2.96 | -0.57 | 0.979 |
| Latency to enter zone | Pooled | Control / Silver vine-J | 1.07 | 0.49 | 0.31 | 3.72 | -0.03 | 1.000 |
| Latency to enter zone | Pooled | Control / Silver vine-A | 0.91 | 0.43 | 0.25 | 3.34 | -0.37 | 0.996 |
| Latency to enter zone | Pooled | Fishmint / Silver vine-C | 0.74 | 0.33 | 0.22 | 2.51 | -0.87 | 0.909 |
| Latency to enter zone | Pooled | Fishmint / Silver vine-J | 0.95 | 0.42 | 0.28 | 3.21 | -0.31 | 0.998 |
| Latency to enter zone | Pooled | Fishmint / Silver vine-A | 0.81 | 0.38 | 0.23 | 2.88 | -0.64 | 0.968 |
| Latency to enter zone | Pooled | Silver vine-C / Silver vine-J | 1.40 | 0.58 | 0.45 | 4.36 | 0.60 | 0.975 |
| Latency to enter zone | Pooled | Silver vine-C / Silver vine-A | 1.19 | 0.52 | 0.36 | 3.89 | 0.20 | 1.000 |
| Latency to enter zone | Pooled | Silver vine-J / Silver vine-A | 0.93 | 0.40 | 0.28 | 3.04 | -0.37 | 0.996 |
| Latency to enter zone | August | Control / Fishmint | 0.85 | 0.45 | 0.20 | 3.63 | -1.46 | 0.586 |
| Latency to enter zone | August | Control / Silver vine-C | 2.03 | 1.02 | 0.51 | 8.02 | 0.18 | 1.000 |
| Latency to enter zone | August | Control / Silver vine-J | 2.06 | 1.22 | 0.41 | 10.42 | 0.18 | 1.000 |
| Latency to enter zone | August | Control / Silver vine-A | 1.67 | 0.85 | 0.42 | 6.65 | -0.21 | 1.000 |
| Latency to enter zone | August | Fishmint / Silver vine-C | 4.41 | 2.30 | 1.07 | 18.25 | 1.66 | 0.457 |
| Latency to enter zone | August | Fishmint / Silver vine-J | 4.48 | 2.68 | 0.87 | 22.96 | 1.47 | 0.581 |
| Latency to enter zone | August | Fishmint / Silver vine-A | 3.62 | 1.86 | 0.89 | 14.68 | 1.31 | 0.688 |
| Latency to enter zone | August | Silver vine-C / Silver vine-J | 1.89 | 0.93 | 0.49 | 7.18 | 0.03 | 1.000 |
| Latency to enter zone | August | Silver vine-C / Silver vine-A | 1.52 | 0.70 | 0.44 | 5.33 | -0.43 | 0.993 |
| Latency to enter zone | August | Silver vine-J / Silver vine-A | 1.50 | 0.78 | 0.36 | 6.19 | -0.41 | 0.994 |
| Latency to enter zone | November | Control / Fishmint | 5.99 | 4.23 | 0.87 | 41.10 | 1.06 | 0.826 |
| Latency to enter zone | November | Control / Silver vine-C | 11.46 | 9.01 | 1.34 | 97.90 | 1.78 | 0.385 |
| Latency to enter zone | November | Control / Silver vine-J | 7.44 | 5.81 | 0.88 | 62.70 | 1.24 | 0.729 |
| Latency to enter zone | November | Control / Silver vine-A | 7.22 | 5.82 | 0.80 | 65.00 | 1.16 | 0.772 |
| Latency to enter zone | November | Fishmint / Silver vine-C | 5.41 | 4.33 | 0.61 | 48.00 | 0.81 | 0.927 |
| Latency to enter zone | November | Fishmint / Silver vine-J | 3.51 | 2.81 | 0.40 | 31.10 | 0.27 | 0.999 |
| Latency to enter zone | November | Fishmint / Silver vine-A | 3.41 | 2.85 | 0.35 | 33.50 | 0.22 | 0.999 |
| Latency to enter zone | November | Silver vine-C / Silver vine-J | 1.83 | 1.62 | 0.17 | 20.30 | -0.49 | 0.988 |
| Latency to enter zone | November | Silver vine-C / Silver vine-A | 1.78 | 1.57 | 0.16 | 19.70 | -0.53 | 0.985 |
| Latency to enter zone | November | Silver vine-J / Silver vine-A | 2.74 | 2.47 | 0.24 | 31.90 | -0.03 | 1.000 |
| Latency to interact | Pooled | Control / Fishmint | 1.32 | 0.90 | 0.21 | 8.42 | 0.41 | 0.994 |
| Latency to interact | Pooled | Control / Silver vine-C | 0.90 | 0.57 | 0.16 | 5.02 | -0.17 | 1.000 |
| Latency to interact | Pooled | Control / Silver vine-J | 1.33 | 0.87 | 0.23 | 7.85 | 0.44 | 0.993 |
| Latency to interact | Pooled | Control / Silver vine-A | 1.17 | 0.77 | 0.20 | 7.01 | 0.24 | 0.999 |
| Latency to interact | Pooled | Fishmint / Silver vine-C | 0.68 | 0.31 | 0.20 | 2.38 | -0.84 | 0.920 |
| Latency to interact | Pooled | Fishmint / Silver vine-J | 1.01 | 0.49 | 0.27 | 3.81 | 0.01 | 1.000 |
| Latency to interact | Pooled | Fishmint / Silver vine-A | 0.89 | 0.44 | 0.23 | 3.41 | -0.24 | 0.999 |
| Latency to interact | Pooled | Silver vine-C / Silver vine-J | 1.48 | 0.61 | 0.47 | 4.59 | 0.94 | 0.883 |
| Latency to interact | Pooled | Silver vine-C / Silver vine-A | 1.30 | 0.55 | 0.41 | 4.08 | 0.63 | 0.971 |
| Latency to interact | Pooled | Silver vine-J / Silver vine-A | 0.88 | 0.40 | 0.26 | 3.04 | -0.28 | 0.999 |
| Latency to interact | August | Control / Fishmint | 0.69 | 0.56 | 0.08 | 6.16 | -0.58 | 0.978 |
| Latency to interact | August | Control / Silver vine-C | 1.65 | 1.38 | 0.17 | 16.06 | 0.48 | 0.989 |
| Latency to interact | August | Control / Silver vine-J | 2.82 | 2.42 | 0.27 | 29.26 | 1.09 | 0.811 |
| Latency to interact | August | Control / Silver vine-A | 0.35 | 0.30 | 0.03 | 3.61 | -1.34 | 0.666 |
| Latency to interact | August | Fishmint / Silver vine-C | 2.62 | 1.58 | 0.51 | 13.61 | 1.43 | 0.606 |
| Latency to interact | August | Fishmint / Silver vine-J | 4.49 | 2.85 | 0.79 | 25.46 | 2.20 | 0.179 |
| Latency to interact | August | Fishmint / Silver vine-A | 0.56 | 0.34 | 0.11 | 2.96 | -1.11 | 0.800 |
| Latency to interact | August | Silver vine-C / Silver vine-J | 1.89 | 1.02 | 0.43 | 8.30 | 0.99 | 0.861 |
| **Latency to interact** | **August** | **Silver vine-C / Silver vine-A** | **0.24** | **0.12** | **0.06** | **0.97** | **-2.98** | **0.024** |
| **Latency to interact** | **August** | **Silver vine-J / Silver vine-A** | **0.14** | **0.07** | **0.03** | **0.60** | **-3.88** | **0.001** |
| Latency to interact | November | Control / Fishmint | 4.12 | 3.52 | 0.40 | 42.24 | 1.40 | 0.629 |
| Latency to interact | November | Control / Silver vine-C | 1.05 | 0.77 | 0.14 | 7.79 | -0.24 | 0.999 |
| Latency to interact | November | Control / Silver vine-J | 2.23 | 1.82 | 0.24 | 20.77 | 0.70 | 0.956 |
| Latency to interact | November | Control / Silver vine-A | 4.75 | 3.98 | 0.48 | 46.85 | 1.59 | 0.506 |
| Latency to interact | November | Fishmint / Silver vine-C | 0.32 | 0.18 | 0.07 | 1.46 | -2.45 | 0.103 |
| Latency to interact | November | Fishmint / Silver vine-J | 0.68 | 0.39 | 0.14 | 3.31 | -1.06 | 0.827 |
| Latency to interact | November | Fishmint / Silver vine-A | 1.44 | 0.81 | 0.31 | 6.63 | 0.25 | 0.999 |
| Latency to interact | November | Silver vine-C / Silver vine-J | 2.66 | 1.31 | 0.69 | 10.22 | 1.53 | 0.545 |
| **Latency to interact** | **November** | **Silver vine-C / Silver vine-A** | **5.67** | **2.95** | **1.37** | **23.42** | **2.91** | **0.030** |
| Latency to interact | November | Silver vine-J / Silver vine-A | 2.67 | 1.40 | 0.64 | 11.13 | 1.45 | 0.597 |
| Active duration | Pooled | Control / Fishmint | 1.07 | 0.24 | 0.58 | 1.98 | 0.12 | 1.000 |
| Active duration | Pooled | Control / Silver vine-C | 0.82 | 0.17 | 0.46 | 1.46 | -1.14 | 0.784 |
| Active duration | Pooled | Control / Silver vine-J | 0.89 | 0.19 | 0.49 | 1.60 | -0.73 | 0.949 |
| Active duration | Pooled | Control / Silver vine-A | 0.92 | 0.20 | 0.51 | 1.67 | -0.57 | 0.979 |
| Active duration | Pooled | Fishmint / Silver vine-C | 0.79 | 0.17 | 0.44 | 1.42 | -1.27 | 0.712 |
| Active duration | Pooled | Fishmint / Silver vine-J | 0.86 | 0.19 | 0.48 | 1.56 | -0.86 | 0.913 |
| Active duration | Pooled | Fishmint / Silver vine-A | 0.89 | 0.20 | 0.49 | 1.63 | -0.69 | 0.958 |
| Active duration | Pooled | Silver vine-C / Silver vine-J | 1.13 | 0.23 | 0.65 | 1.98 | 0.41 | 0.994 |
| Active duration | Pooled | Silver vine-C / Silver vine-A | 1.17 | 0.24 | 0.67 | 2.06 | 0.57 | 0.979 |
| Active duration | Pooled | Silver vine-J / Silver vine-A | 1.08 | 0.23 | 0.61 | 1.91 | 0.16 | 1.000 |
| Active duration | August | Control / Fishmint | 1.64 | 0.62 | 0.59 | 4.56 | 0.64 | 0.969 |
| Active duration | August | Control / Silver vine-C | 0.82 | 0.27 | 0.33 | 2.00 | -1.41 | 0.624 |
| Active duration | August | Control / Silver vine-J | 1.02 | 0.35 | 0.40 | 2.60 | -0.69 | 0.960 |
| Active duration | August | Control / Silver vine-A | 0.99 | 0.34 | 0.39 | 2.49 | -0.79 | 0.933 |
| Active duration | August | Fishmint / Silver vine-C | 0.64 | 0.22 | 0.26 | 1.62 | -2.07 | 0.236 |
| Active duration | August | Fishmint / Silver vine-J | 0.81 | 0.28 | 0.31 | 2.10 | -1.35 | 0.662 |
| Active duration | August | Fishmint / Silver vine-A | 0.78 | 0.28 | 0.29 | 2.07 | -1.42 | 0.615 |
| Active duration | August | Silver vine-C / Silver vine-J | 1.62 | 0.49 | 0.72 | 3.69 | 0.76 | 0.943 |
| Active duration | August | Silver vine-C / Silver vine-A | 1.57 | 0.48 | 0.68 | 3.63 | 0.62 | 0.971 |
| Active duration | August | Silver vine-J / Silver vine-A | 1.25 | 0.40 | 0.52 | 3.00 | -0.11 | 1.000 |
| Active duration | November | Control / Fishmint | 0.91 | 0.19 | 0.52 | 1.61 | -0.44 | 0.992 |
| Active duration | November | Control / Silver vine-C | 0.95 | 0.20 | 0.53 | 1.68 | -0.27 | 0.999 |
| Active duration | November | Control / Silver vine-J | 0.91 | 0.19 | 0.52 | 1.60 | -0.46 | 0.991 |
| Active duration | November | Control / Silver vine-A | 0.96 | 0.20 | 0.54 | 1.71 | -0.17 | 1.000 |
| Active duration | November | Fishmint / Silver vine-C | 1.04 | 0.21 | 0.59 | 1.81 | 0.18 | 1.000 |
| Active duration | November | Fishmint / Silver vine-J | 1.00 | 0.20 | 0.57 | 1.73 | -0.02 | 1.000 |
| Active duration | November | Fishmint / Silver vine-A | 1.06 | 0.22 | 0.60 | 1.85 | 0.27 | 0.999 |
| Active duration | November | Silver vine-C / Silver vine-J | 0.96 | 0.20 | 0.55 | 1.68 | -0.20 | 1.000 |
| Active duration | November | Silver vine-C / Silver vine-A | 1.02 | 0.21 | 0.58 | 1.80 | 0.09 | 1.000 |
| Active duration | November | Silver vine-J / Silver vine-A | 1.06 | 0.22 | 0.61 | 1.86 | 0.29 | 0.998 |
| Zone Duration | Pooled | Control / Fishmint | 1.03 | 0.42 | 0.34 | 3.15 | -1.31 | 0.685 |
| Zone Duration | Pooled | Control / Silver vine-C | 0.65 | 0.25 | 0.22 | 1.87 | -2.58 | 0.075 |
| Zone Duration | Pooled | Control / Silver vine-J | 0.94 | 0.38 | 0.32 | 2.82 | -1.55 | 0.528 |
| Zone Duration | Pooled | Control / Silver vine-A | 2.42 | 1.10 | 0.70 | 8.36 | 0.70 | 0.958 |
| Zone Duration | Pooled | Fishmint / Silver vine-C | 1.10 | 0.39 | 0.42 | 2.88 | -1.33 | 0.672 |
| Zone Duration | Pooled | Fishmint / Silver vine-J | 1.61 | 0.60 | 0.59 | 4.43 | -0.24 | 0.999 |
| Zone Duration | Pooled | Fishmint / Silver vine-A | 4.13 | 1.76 | 1.29 | 13.20 | 2.00 | 0.266 |
| Zone Duration | Pooled | Silver vine-C / Silver vine-J | 2.58 | 0.90 | 1.00 | 6.65 | 1.10 | 0.809 |
| **Zone Duration** | **Pooled** | **Silver vine-C / Silver vine-A** | **6.59** | **2.68** | **2.17** | **19.97** | **3.25** | **0.010** |
| Zone Duration | Pooled | Silver vine-J / Silver vine-A | 4.50 | 1.90 | 1.42 | 14.25 | 2.22 | 0.171 |
| Zone Duration | August | Control / Fishmint | 1.16 | 0.72 | 0.21 | 6.35 | -0.02 | 1.000 |
| Zone Duration | August | Control / Silver vine-C | 0.90 | 0.54 | 0.18 | 4.55 | -0.45 | 0.992 |
| Zone Duration | August | Control / Silver vine-J | 0.91 | 0.54 | 0.18 | 4.59 | -0.44 | 0.992 |
| Zone Duration | August | Control / Silver vine-A | 1.56 | 1.02 | 0.26 | 9.32 | 0.43 | 0.993 |
| Zone Duration | August | Fishmint / Silver vine-C | 0.91 | 0.54 | 0.18 | 4.54 | -0.43 | 0.993 |
| Zone Duration | August | Fishmint / Silver vine-J | 0.92 | 0.54 | 0.18 | 4.57 | -0.42 | 0.993 |
| Zone Duration | August | Fishmint / Silver vine-A | 1.57 | 1.03 | 0.26 | 9.41 | 0.45 | 0.992 |
| Zone Duration | August | Silver vine-C / Silver vine-J | 1.18 | 0.66 | 0.26 | 5.41 | 0.01 | 1.000 |
| Zone Duration | August | Silver vine-C / Silver vine-A | 2.03 | 1.28 | 0.37 | 11.27 | 0.87 | 0.908 |
| Zone Duration | August | Silver vine-J / Silver vine-A | 2.02 | 1.27 | 0.36 | 11.25 | 0.86 | 0.912 |
| Zone Duration | November | Control / Fishmint | 0.67 | 0.37 | 0.15 | 2.96 | -1.61 | 0.493 |
| Zone Duration | November | Control / Silver vine-C | 0.39 | 0.21 | 0.09 | 1.63 | -2.70 | 0.054 |
| Zone Duration | November | Control / Silver vine-J | 0.61 | 0.33 | 0.14 | 2.62 | -1.81 | 0.366 |
| Zone Duration | November | Control / Silver vine-A | 1.96 | 1.19 | 0.37 | 10.35 | 0.32 | 0.998 |
| Zone Duration | November | Fishmint / Silver vine-C | 0.93 | 0.43 | 0.27 | 3.24 | -1.19 | 0.756 |
| Zone Duration | November | Fishmint / Silver vine-J | 1.46 | 0.70 | 0.40 | 5.42 | -0.20 | 1.000 |
| Zone Duration | November | Fishmint / Silver vine-A | 4.70 | 2.65 | 1.01 | 21.85 | 1.91 | 0.314 |
| Zone Duration | November | Silver vine-C / Silver vine-J | 2.52 | 1.15 | 0.73 | 8.71 | 0.99 | 0.861 |
| **Zone Duration** | **November** | **Silver vine-C / Silver vine-A** | **8.09** | **4.38** | **1.85** | **35.46** | **2.98** | **0.024** |
| Zone Duration | November | Silver vine-J / Silver vine-A | 5.16 | 2.85 | 1.15 | 23.26 | 2.12 | 0.214 |
| Zone duration (offset) | Pooled | Control / Fishmint | 1.17 | 0.49 | 0.37 | 3.67 | -0.76 | 0.942 |
| Zone duration (offset) | Pooled | Control / Silver vine-C | 0.80 | 0.32 | 0.27 | 2.37 | -1.75 | 0.405 |
| Zone duration (offset) | Pooled | Control / Silver vine-J | 1.24 | 0.51 | 0.41 | 3.78 | -0.63 | 0.970 |
| Zone duration (offset) | Pooled | Control / Silver vine-A | 2.50 | 1.15 | 0.71 | 8.76 | 0.97 | 0.870 |
| Zone duration (offset) | Pooled | Fishmint / Silver vine-C | 1.11 | 0.41 | 0.41 | 3.00 | -1.02 | 0.848 |
| Zone duration (offset) | Pooled | Fishmint / Silver vine-J | 1.71 | 0.66 | 0.60 | 4.87 | 0.16 | 1.000 |
| Zone duration (offset) | Pooled | Fishmint / Silver vine-A | 3.44 | 1.51 | 1.04 | 11.35 | 1.75 | 0.406 |
| Zone duration (offset) | Pooled | Silver vine-C / Silver vine-J | 2.48 | 0.89 | 0.93 | 6.59 | 1.21 | 0.747 |
| **Zone duration (offset)** | **Pooled** | **Silver vine-C / Silver vine-A** | **5.00** | **2.07** | **1.61** | **15.47** | **2.74** | **0.048** |
| Zone duration (offset) | Pooled | Silver vine-J / Silver vine-A | 3.24 | 1.39 | 1.00 | 10.48 | 1.63 | 0.477 |
| Zone duration (offset) | August | Control / Fishmint | 1.00 | 0.59 | 0.20 | 5.04 | -0.06 | 1.000 |
| Zone duration (offset) | August | Control / Silver vine-C | 1.12 | 0.61 | 0.26 | 4.91 | 0.14 | 1.000 |
| Zone duration (offset) | August | Control / Silver vine-J | 1.05 | 0.58 | 0.24 | 4.70 | 0.02 | 1.000 |
| Zone duration (offset) | August | Control / Silver vine-A | 1.65 | 1.01 | 0.31 | 8.78 | 0.76 | 0.942 |
| Zone duration (offset) | August | Fishmint / Silver vine-C | 1.16 | 0.66 | 0.25 | 5.46 | 0.20 | 1.000 |
| Zone duration (offset) | August | Fishmint / Silver vine-J | 1.09 | 0.63 | 0.22 | 5.30 | 0.08 | 1.000 |
| Zone duration (offset) | August | Fishmint / Silver vine-A | 1.71 | 1.07 | 0.31 | 9.47 | 0.80 | 0.931 |
| Zone duration (offset) | August | Silver vine-C / Silver vine-J | 0.97 | 0.51 | 0.24 | 4.01 | -0.12 | 1.000 |
| Zone duration (offset) | August | Silver vine-C / Silver vine-A | 1.53 | 0.90 | 0.31 | 7.62 | 0.66 | 0.965 |
| Zone duration (offset) | August | Silver vine-J / Silver vine-A | 1.63 | 0.98 | 0.32 | 8.33 | 0.76 | 0.942 |
| Zone duration (offset) | November | Control / Fishmint | 0.83 | 0.46 | 0.18 | 3.82 | -1.18 | 0.762 |
| Zone duration (offset) | November | Control / Silver vine-C | 0.49 | 0.27 | 0.11 | 2.17 | -2.18 | 0.189 |
| Zone duration (offset) | November | Control / Silver vine-J | 0.79 | 0.43 | 0.18 | 3.52 | -1.30 | 0.692 |
| Zone duration (offset) | November | Control / Silver vine-A | 1.92 | 1.19 | 0.36 | 10.38 | 0.29 | 0.999 |
| Zone duration (offset) | November | Fishmint / Silver vine-C | 0.96 | 0.45 | 0.27 | 3.44 | -1.11 | 0.802 |
| Zone duration (offset) | November | Fishmint / Silver vine-J | 1.53 | 0.75 | 0.40 | 5.79 | -0.11 | 1.000 |
| Zone duration (offset) | November | Fishmint / Silver vine-A | 3.72 | 2.12 | 0.78 | 17.66 | 1.47 | 0.582 |
| Zone duration (offset) | November | Silver vine-C / Silver vine-J | 2.57 | 1.20 | 0.72 | 9.20 | 1.00 | 0.854 |
| Zone duration (offset) | November | Silver vine-C / Silver vine-A | 6.27 | 3.46 | 1.39 | 28.21 | 2.47 | 0.098 |
| Zone duration (offset) | November | Silver vine-J / Silver vine-A | 3.92 | 2.19 | 0.86 | 17.94 | 1.60 | 0.499 |
| Interaction duration | Pooled | Control / Fishmint | 0.33 | 0.24 | 0.05 | 2.33 | -2.67 | 0.058 |
| **Interaction duration** | **Pooled** | **Control / Silver vine-C** | **0.09** | **0.06** | **0.01** | **0.58** | **-4.72** | **<0.001** |
| **Interaction duration** | **Pooled** | **Control / Silver vine-J** | **0.26** | **0.18** | **0.04** | **1.74** | **-3.08** | **0.018** |
| Interaction duration | Pooled | Control / Silver vine-A | 0.63 | 0.47 | 0.08 | 4.91 | -1.68 | 0.445 |
| **Interaction duration** | **Pooled** | **Fishmint / Silver vine-C** | **0.63** | **0.29** | **0.18** | **2.16** | **-2.80** | **0.041** |
| Interaction duration | Pooled | Fishmint / Silver vine-J | 1.74 | 0.85 | 0.46 | 6.62 | -0.51 | 0.987 |
| Interaction duration | Pooled | Fishmint / Silver vine-A | 4.27 | 2.38 | 0.93 | 19.52 | 1.16 | 0.774 |
| Interaction duration | Pooled | Silver vine-C / Silver vine-J | 6.17 | 2.65 | 1.91 | 19.92 | 2.37 | 0.125 |
| **Interaction duration** | **Pooled** | **Silver vine-C / Silver vine-A** | **15.09** | **7.56** | **3.85** | **59.18** | **3.81** | **0.001** |
| Interaction duration | Pooled | Silver vine-J / Silver vine-A | 5.46 | 2.97 | 1.24 | 24.03 | 1.65 | 0.467 |
| Interaction duration | August | Control / Fishmint | 0.06 | 0.10 | 0.00 | 6.26 | -1.86 | 0.338 |
| Interaction duration | August | Control / Silver vine-C | 0.04 | 0.07 | 0.00 | 4.62 | -2.03 | 0.251 |
| Interaction duration | August | Control / Silver vine-J | 0.04 | 0.06 | 0.00 | 3.73 | -2.15 | 0.198 |
| Interaction duration | August | Control / Silver vine-A | 0.09 | 0.16 | 0.00 | 10.76 | -1.57 | 0.518 |
| Interaction duration | August | Fishmint / Silver vine-C | 1.07 | 0.68 | 0.19 | 6.14 | -0.42 | 0.994 |
| Interaction duration | August | Fishmint / Silver vine-J | 0.88 | 0.55 | 0.16 | 4.78 | -0.74 | 0.947 |
| Interaction duration | August | Fishmint / Silver vine-A | 2.21 | 1.68 | 0.28 | 17.54 | 0.61 | 0.974 |
| Interaction duration | August | Silver vine-C / Silver vine-J | 1.15 | 0.67 | 0.24 | 5.63 | -0.33 | 0.998 |
| Interaction duration | August | Silver vine-C / Silver vine-A | 2.90 | 2.09 | 0.41 | 20.62 | 1.02 | 0.848 |
| Interaction duration | August | Silver vine-J / Silver vine-A | 3.51 | 2.46 | 0.52 | 23.81 | 1.31 | 0.684 |
| Interaction duration | November | Control / Fishmint | 0.41 | 0.35 | 0.04 | 4.14 | -1.75 | 0.403 |
| **Interaction duration** | **November** | **Control / Silver vine-C** | **0.08** | **0.06** | **0.01** | **0.67** | **-3.98** | **0.001** |
| Interaction duration | November | Control / Silver vine-J | 0.35 | 0.30 | 0.04 | 3.47 | -1.95 | 0.293 |
| Interaction duration | November | Control / Silver vine-A | 0.61 | 0.55 | 0.05 | 7.06 | -1.21 | 0.748 |
| **Interaction duration** | **November** | **Fishmint / Silver vine-C** | **0.34** | **0.20** | **0.07** | **1.75** | **-2.78** | **0.043** |
| Interaction duration | November | Fishmint / Silver vine-J | 1.56 | 1.05 | 0.25 | 9.77 | -0.21 | 1.000 |
| Interaction duration | November | Fishmint / Silver vine-A | 2.71 | 1.97 | 0.37 | 19.69 | 0.56 | 0.980 |
| Interaction duration | November | Silver vine-C / Silver vine-J | 8.30 | 4.93 | 1.64 | 41.94 | 2.58 | 0.075 |
| **Interaction duration** | **November** | **Silver vine-C / Silver vine-A** | **14.38** | **9.36** | **2.44** | **84.83** | **3.20** | **0.012** |
| Interaction duration | November | Silver vine-J / Silver vine-A | 3.12 | 2.23 | 0.44 | 21.90 | 0.77 | 0.940 |
| Interaction duration (offset) | Pooled | Control / Fishmint | 0.36 | 0.25 | 0.06 | 2.41 | -2.41 | 0.112 |
| **Interaction duration (offset)** | **Pooled** | **Control / Silver vine-C** | **0.11** | **0.07** | **0.02** | **0.62** | **-4.48** | **<0.001** |
| Interaction duration (offset) | Pooled | Control / Silver vine-J | 0.33 | 0.22 | 0.05 | 2.09 | -2.61 | 0.068 |
| Interaction duration (offset) | Pooled | Control / Silver vine-A | 0.63 | 0.46 | 0.09 | 4.60 | -1.54 | 0.540 |
| **Interaction duration (offset)** | **Pooled** | **Fishmint / Silver vine-C** | **0.57** | **0.25** | **0.17** | **1.91** | **-2.75** | **0.047** |
| Interaction duration (offset) | Pooled | Fishmint / Silver vine-J | 1.76 | 0.85 | 0.47 | 6.55 | -0.20 | 1.000 |
| Interaction duration (offset) | Pooled | Fishmint / Silver vine-A | 3.36 | 1.85 | 0.75 | 15.13 | 1.00 | 0.854 |
| Interaction duration (offset) | Pooled | Silver vine-C / Silver vine-J | 5.97 | 2.49 | 1.91 | 18.62 | 2.70 | 0.053 |
| **Interaction duration (offset)** | **Pooled** | **Silver vine-C / Silver vine-A** | **11.43** | **5.61** | **3.00** | **43.58** | **3.62** | **0.003** |
| Interaction duration (offset) | Pooled | Silver vine-J / Silver vine-A | 3.70 | 1.97 | 0.87 | 15.81 | 1.22 | 0.740 |
| Interaction duration (offset) | August | Control / Fishmint | 0.04 | 0.06 | 0.00 | 3.95 | -2.01 | 0.259 |
| Interaction duration (offset) | August | Control / Silver vine-C | 0.05 | 0.08 | 0.00 | 4.57 | -1.92 | 0.305 |
| Interaction duration (offset) | August | Control / Silver vine-J | 0.04 | 0.06 | 0.00 | 3.46 | -2.09 | 0.226 |
| Interaction duration (offset) | August | Control / Silver vine-A | 0.08 | 0.14 | 0.00 | 8.81 | -1.56 | 0.522 |
| Interaction duration (offset) | August | Fishmint / Silver vine-C | 1.41 | 0.86 | 0.27 | 7.46 | 0.30 | 0.998 |
| Interaction duration (offset) | August | Fishmint / Silver vine-J | 1.08 | 0.64 | 0.22 | 5.46 | -0.14 | 1.000 |
| Interaction duration (offset) | August | Fishmint / Silver vine-A | 2.43 | 1.75 | 0.34 | 17.24 | 1.02 | 0.849 |
| Interaction duration (offset) | August | Silver vine-C / Silver vine-J | 0.91 | 0.49 | 0.20 | 4.01 | -0.48 | 0.989 |
| Interaction duration (offset) | August | Silver vine-C / Silver vine-A | 2.03 | 1.38 | 0.32 | 12.88 | 0.81 | 0.928 |
| Interaction duration (offset) | August | Silver vine-J / Silver vine-A | 2.64 | 1.75 | 0.43 | 16.05 | 1.22 | 0.739 |
| Interaction duration (offset) | November | Control / Fishmint | 0.60 | 0.48 | 0.07 | 5.34 | -1.37 | 0.648 |
| **Interaction duration (offset)** | **November** | **Control / Silver vine-C** | **0.11** | **0.08** | **0.01** | **0.80** | **-3.84** | **0.001** |
| Interaction duration (offset) | November | Control / Silver vine-J | 0.55 | 0.43 | 0.06 | 4.69 | -1.51 | 0.557 |
| Interaction duration (offset) | November | Control / Silver vine-A | 0.72 | 0.60 | 0.07 | 7.09 | -1.09 | 0.810 |
| **Interaction duration (offset)** | **November** | **Fishmint / Silver vine-C** | **0.32** | **0.18** | **0.07** | **1.48** | **-3.07** | **0.018** |
| Interaction duration (offset) | November | Fishmint / Silver vine-J | 1.65 | 1.04 | 0.30 | 9.22 | -0.14 | 1.000 |
| Interaction duration (offset) | November | Fishmint / Silver vine-A | 2.15 | 1.48 | 0.33 | 14.13 | 0.26 | 0.999 |
| **Interaction duration (offset)** | **November** | **Silver vine-C / Silver vine-J** | **9.33** | **5.06** | **2.13** | **40.94** | **3.04** | **0.020** |
| **Interaction duration (offset)** | **November** | **Silver vine-C / Silver vine-A** | **12.17** | **7.41** | **2.31** | **64.08** | **3.14** | **0.015** |
| Interaction duration (offset) | November | Silver vine-J / Silver vine-A | 2.35 | 1.57 | 0.38 | 14.61 | 0.40 | 0.995 |

**Supplementary Table S6** Chemical analyses of four plant products showing peak areas from GC-MS chromatographs (±SD, n=3 replicates for each analysis). In bold are identified compounds with known or purported stimulating effect on cats (Bol et al. 2017; Lichman et al. 2020; Tucker and Tucker 1988).

| Peak | RT (min)† | Name | Fishmint | Silver vine C | Silver vine J | Silver vine A |
| --- | --- | --- | --- | --- | --- | --- |
| **a. Headspace solid-phase microextraction (HS-SPME)** | | | | |  |  |
| 1 | 3.00 | Undecane, 3,6-dimethyl- | 868 ± 271 | 1054 ± 483 | 1608817 ± 47534 | 5098 ± 871 |
| 2 | 3.58 | Dodecane, 2,6,10-trimethyl- | 130 ± 40 | 95 ± 165 | 1356698 ± 130860 | 3086 ± 2330 |
| 3 | 5.20 | D-Limonene | 3926 ± 471 | 56407 ± 15198 | 180090 ± 10414 | 762735 ± 90827 |
| 4 | 8.37 | Unknown1 | 71 ± 106 | 2240426 ± 368829 | 5344572 ± 77076 | 6075888 ± 117227 |
| 5 | 9.02 | Unknown2 | 485 ± 779 | 1709715 ± 399968 | 1171610 ± 110720 | 1020869 ± 68247 |
| 6 | 9.92 | Ethanone, 1-(1-cyclohexen-1-yl)- | 1869 ± 2833 | 215710 ± 85266 | 348575 ± 26765 | 2210252 ± 259654 |
| 7 | 10.13 | Acetic acid | 476186 ± 626017 | 24491906 ± 3148887 | 32976144 ± 17716930 | 20981991 ± 2373620 |
| 8 | 11.28 | Unknown3 | 585 ± 265 | 760569 ± 371442 | 1047076 ± 21535 | 1606912 ± 184291 |
| 9 | 12.91 | 4-Isopropylcyclohexa-1,3-dienecarbaldehyde | 991 ± 296 | 5082787 ± 450818 | 2093307 ± 210596 | 4547217 ± 413882 |
| 10 | 14.03 | Caryophyllene-RI1419 | 128 ± 57 | 2759 ± 767 | 2254 ± 614 | 18037457 ± 1037935 |
| 11 | 15.22 | Hexanoic acid | 1205842 ± 104861 | 4855207 ± 734837 | 3387049 ± 212153 | 3043284 ± 281507 |
| 12 | 16.47 | 4,4-Dimethyl-cyclohex-2-en-1-ol | 1993 ± 1639 | 1011453 ± 173203 | 136365 ± 67520 | 51196 ± 8238 |
| 13 | 17.40 | Borane, ethylisopropylmethyl- | 21962 ± 31968 | 615839 ± 73631 | 303393 ± 125162 | 178157 ± 8654 |
| 14 | 18.04 | Dill ether | 7326 ± 2876 | 14440403 ± 1740492 | 7755721 ± 640708 | 9975868 ± 617766 |
| **15†** | **19.31** | **cis-cis-Nepetalactone** | **8 ± 14** | **86511 ± 12929** | **38241 ± 3676** | **16703929 ± 1378247** |
| **16** | **20.01** | **cis-trans-Nepetalactone** | **21087 ± 10575** | **28675 ± 18697** | **3946 ± 635** | **48497248 ± 3148073** |
| **17†** | **20.96** | **(+)-Isodihydronepetalactone** | **535 ± 705** | **2204834 ± 388445** | **1572667 ± 137248** | **3176894 ± 370183** |
| **18** | **21.65** | **Iridomyrmecin** | **6187 ± 3187** | **579904 ± 379042** | **302463 ± 153836** | **1306204 ± 457614** |
| **19†** | **21.83** | **(+)-Dihydronepetalactone** | **518 ± 11** | **109040 ± 83757** | **25506 ± 2784** | **220414 ± 34100** |
| 20† | 22.76 | 3-Oxabicyclo[5.3.0]decan-2-one, 9-methylene-, trans- | 1201 ± 365 | 928074 ± 113843 | 990975 ± 111711 | 773409 ± 102231 |
|  |  |  |  |  |  |  |
| **b. Solvent extraction** | | |  |  |  |  |
| **21** | **22.58** | **Actinidine** | **30790 ± 7184** | **4508851 ± 1141521** | **3936193 ± 2913865** | **2590283 ± 393310** |
| **22†** | **24.27** | **cis-cis-Nepetalactone** | **2150 ± 2161** | **21338 ± 4087** | **3499 ± 1478** | **1772031 ± 1260822** |
| **23†** | **24.97** | **(+)-Isodihydronepetalactone** | **204 ± 288** | **198158 ± 81799** | **224520 ± 122170** | **216725 ± 80836** |
| **24†** | **26.12** | **(+)-Dihydronepetalactone** | **3513 ± 3019** | **48018 ± 18133** | **54781 ± 32760** | **40740 ± 16944** |
| 25† | 27.09 | 3-Oxabicyclo[5.3.0]decan-2-one, 9-methylene-, trans- | 3024 ± 2806 | 170206 ± 76927 | 223285 ± 72187 | 123246 ± 33385 |
| 26 | 30.10 | 2H-Oxecin-2-one, 3,4,7,8,9,10-hexahydro-4-hydroxy-10-methyl-, [4S-( 4R*,5E,10S*)]- | 1850 ± 185 | 111258 ± 33102 | 140763 ± 96929 | 127867 ± 19880 |
| 27 | 37.05 | Palmitic acid | 0 ± 0 | 90395 ± 47690 | 236110 ± 351370 | 155900 ± 88230 |
| 28 | 46.57 | Phenol | 597877 ± 987686 | 932143 ± 1480288 | 1309663 ± 2112597 | 716088 ± 1124808 |
| 29 | 49.05 | Unknown-RT49.05 | 438582 ± 387293 | 706437 ± 616896 | 688156 ± 674119 | 754765 ± 646730 |

**Supplementary Table S7** Eigenvalues, percentage of variance explained, and cumulative percentage of variance explained for each principal component (PC) and eigenvectors for (a) HS-SPME extraction and (b) solvent extraction.

| **a. Headspace solid-phase microextraction (HS-SPME)** | | **PC1** | **PC2** | **PC3** | **PC4** | **PC5** | **PC6** | **PC7** | **PC8** | **PC9** | **PC10** | **PC11** | **PC12** |
| --- | --- | --- | --- | --- | --- | --- | --- | --- | --- | --- | --- | --- | --- |
| Eigenvalue | | 10.44 | 5.73 | 2.92 | 0.56 | 0.19 | 0.08 | 0.05 | 0.02 | 0.01 | <0.01 | <0.01 | <0.01 |
| Variance explained | | 52.22 | 28.63 | 14.62 | 2.81 | 0.95 | 0.38 | 0.25 | 0.11 | 0.03 | 0.01 | <0.01 | <0.01 |
| Cumulative variance explained (%) | | 52.22 | 80.85 | 95.47 | 98.28 | 99.22 | 99.60 | 99.85 | 99.96 | 99.99 | 100.00 | 100.00 | 100.00 |
| Eigenvectors | |  |  |  |  |  |  |  |  |  |  |  |  |
| Peak 1 | Undecane, 3,6-dimethyl- | 0.03 | -0.14 | -0.55 | -0.02 | 0.10 | -0.09 | 0.05 | 0.06 | -0.03 | 0.09 | 0.00 | -0.33 |
| Peak 2 | Dodecane, 2,6,10-trimethyl- | 0.03 | -0.14 | -0.55 | -0.13 | 0.06 | -0.10 | 0.09 | 0.07 | 0.07 | 0.10 | 0.12 | -0.04 |
| Peak 3 | D-Limonene | -0.25 | 0.23 | -0.08 | 0.01 | -0.09 | 0.05 | 0.16 | -0.32 | 0.28 | 0.15 | -0.28 | -0.24 |
| Peak 4 | Unknown1 | -0.25 | 0.03 | -0.35 | 0.01 | -0.16 | -0.10 | -0.18 | -0.30 | -0.17 | -0.22 | -0.02 | 0.31 |
| Peak 5 | Unknown2 | -0.21 | -0.31 | 0.00 | -0.05 | -0.05 | -0.37 | -0.03 | -0.43 | 0.12 | -0.34 | 0.10 | -0.15 |
| Peak 6 | Ethanone, 1-(1-cyclohexen-1-yl)- | -0.25 | 0.23 | -0.03 | 0.00 | -0.17 | -0.09 | 0.27 | -0.28 | 0.22 | 0.20 | -0.09 | 0.25 |
| Peak 7 | Acetic acid | -0.18 | -0.16 | -0.09 | 0.93 | 0.08 | 0.10 | 0.02 | -0.02 | -0.07 | 0.13 | 0.10 | 0.03 |
| Peak 8 | Unknown3 | -0.28 | 0.04 | -0.19 | -0.08 | 0.23 | 0.57 | -0.50 | 0.04 | 0.16 | -0.18 | -0.30 | -0.05 |
| Peak 9 | 4-Isopropylcyclohexa-1,3-dienecarbaldehyde | -0.27 | -0.15 | 0.16 | -0.02 | -0.19 | 0.18 | 0.17 | 0.18 | 0.31 | -0.02 | 0.00 | 0.36 |
| Peak 10 | Caryophyllene-RI1419 | -0.23 | 0.27 | 0.03 | 0.00 | -0.19 | 0.00 | -0.04 | -0.02 | -0.20 | -0.04 | 0.08 | -0.22 |
| Peak 11 | Hexanoic acid | -0.19 | -0.33 | 0.05 | -0.06 | -0.21 | -0.32 | -0.23 | 0.20 | -0.26 | 0.45 | -0.58 | 0.06 |
| Peak 12 | 4,4-Dimethyl-cyclohex-2-en-1-ol | -0.07 | -0.34 | 0.32 | 0.07 | 0.05 | -0.03 | 0.04 | -0.11 | -0.14 | -0.42 | -0.21 | -0.29 |
| Peak 13 | Borane, ethylisopropylmethyl- | -0.12 | -0.36 | 0.12 | -0.25 | 0.04 | 0.43 | 0.03 | -0.42 | -0.32 | 0.44 | 0.34 | -0.02 |
| Peak 14 | Dill ether | -0.24 | -0.25 | 0.09 | -0.04 | -0.16 | 0.05 | -0.01 | 0.23 | 0.38 | 0.02 | 0.17 | -0.20 |
| Peak 15 | cis,cis-Nepetalactone | -0.23 | 0.27 | 0.03 | -0.01 | -0.18 | 0.00 | -0.03 | 0.03 | -0.23 | -0.04 | 0.08 | -0.19 |
| Peak 16 | cis-trans-Nepetalactone | -0.24 | 0.27 | 0.03 | -0.01 | -0.17 | 0.01 | -0.05 | 0.20 | -0.41 | -0.09 | 0.17 | -0.03 |
| Peak 17 | (+)-Isodihydronepetalactone | -0.31 | -0.03 | 0.00 | -0.06 | -0.07 | -0.05 | 0.12 | 0.32 | 0.11 | 0.10 | 0.21 | -0.37 |
| Peak 18 | Iridomyrmecin | -0.28 | 0.10 | 0.07 | -0.08 | 0.66 | -0.04 | 0.48 | 0.04 | -0.18 | 0.00 | -0.24 | -0.02 |
| Peak 19 | (+)-Dihydronepetalactone | -0.28 | 0.09 | 0.15 | -0.06 | 0.46 | -0.40 | -0.44 | 0.03 | 0.14 | 0.15 | 0.33 | 0.18 |
| Peak 20 | 3-Oxabicyclo[5.3.0]decan-2-one, 9-methylene-, trans- | -0.22 | -0.25 | -0.21 | -0.13 | -0.04 | 0.06 | 0.27 | 0.26 | -0.19 | -0.30 | 0.08 | 0.37 |
|  | |  |  |  |  |  |  |  |  |  |  |  |  |
| **b. Solvent extraction** | | **PC1** | **PC2** | **PC3** | **PC4** | **PC5** | **PC6** | **PC7** | **PC8** | **PC9** |  |  |  |
| Eigenvalue | | 5.45 | 2.31 | 0.91 | 0.18 | 0.07 | 0.04 | 0.03 | 0.01 | <0.01 |  |  |  |
| Variance explained | | 60.58 | 25.69 | 10.07 | 1.95 | 0.83 | 0.46 | 0.35 | 0.07 | <0.01 |  |  |  |
| Cumulative variance explained (%) | | 60.58 | 86.26 | 96.33 | 98.28 | 99.11 | 99.57 | 99.92 | 100.00 | 100.00 |  |  |  |
| Eigenvectors | |  |  |  |  |  |  |  |  |  |  |  |  |
| Peak 21 | Actinidine | 0.41 | -0.07 | -0.19 | -0.40 | 0.64 | 0.23 | 0.08 | 0.28 | 0.30 |  |  |  |
| Peak 22 | cis,cis-Nepetalactone | 0.08 | 0.37 | 0.84 | -0.24 | -0.02 | -0.09 | 0.15 | 0.25 | 0.03 |  |  |  |
| Peak 23 | (+)-Isodihydronepetalactone | 0.40 | 0.21 | 0.06 | -0.05 | 0.01 | -0.27 | -0.13 | -0.74 | 0.38 |  |  |  |
| Peak 24 | (+)-Dihydronepetalactone | 0.42 | 0.11 | -0.07 | 0.16 | 0.29 | -0.47 | -0.05 | 0.07 | -0.68 |  |  |  |
| Peak 25 | 2H-Oxecin-2-one, 3,4,7,8,9,10-hexahydro-4-hydroxy-10-methyl-, [4S-(4R*,5E,10S*)]- | 0.42 | -0.06 | 0.10 | -0.18 | -0.32 | 0.57 | -0.49 | -0.05 | -0.31 |  |  |  |
| Peak 26 | 3-Oxabicyclo[5.3.0]decan-2-one, 9-methylene-, trans- | 0.38 | 0.17 | -0.36 | -0.14 | -0.61 | -0.26 | 0.20 | 0.39 | 0.20 |  |  |  |
| Peak 27 | Palmitic acid | 0.37 | -0.22 | 0.23 | 0.79 | 0.02 | 0.13 | 0.02 | 0.20 | 0.29 |  |  |  |
| Peak 28 | Phenol, 2,4-bis(1-methyl-1-phenylethyl)-RI2508 | 0.20 | -0.56 | 0.15 | -0.17 | -0.14 | 0.07 | 0.67 | -0.28 | -0.21 |  |  |  |
| Peak 29 | Unknown-RT49.05 | 0.03 | 0.64 | -0.19 | 0.22 | 0.05 | 0.48 | 0.47 | -0.17 | -0.16 |  |  |  |

**Supplementary Table S8** Results from vector fitting analysis showing how chemical compounds are associated with the PCA ordination of (a) HS-SPME extraction and (b) solvent extraction. Each compound's association with the PC axes, strength of association (r^2^) and significance (p-value) are reported. Compounds with p < 0.05 are considered significantly aligned with the overall ordination.

| Peak | Chemical compounds | PC1 | PC2 | r**^2^** | P-value |
| --- | --- | --- | --- | --- | --- |
| **a. Headspace solid-phase microextraction (HS-SPME)** | |  |  |  |  |
| 1 | Undecane, 3,6-dimethyl- | 0.21 | -0.98 | 0.12 | 0.569 |
| 2 | Dodecane, 2,6,10-trimethyl- | 0.22 | -0.98 | 0.12 | 0.563 |
| 3 | D-Limonene | -0.73 | 0.68 | 0.97 | 0.001 |
| 4 | Unknown1 | -0.99 | 0.12 | 0.63 | 0.020 |
| 5 | Unknown2 | -0.56 | -0.83 | 0.98 | 0.001 |
| 6 | Ethanone, 1-(1-cyclohexen-1-yl)- | -0.74 | 0.68 | 0.99 | 0.001 |
| 7 | Acetic acid | -0.74 | -0.67 | 0.48 | 0.055 |
| 8 | Unknown3 | -0.99 | 0.12 | 0.84 | 0.001 |
| 9 | 4-Isopropylcyclohexa-1,3-dienecarbaldehyde | -0.88 | -0.48 | 0.91 | 0.002 |
| 10 | Caryophyllene-RI1419 | -0.66 | 0.75 | 0.99 | 0.002 |
| 11 | Hexanoic acid | -0.49 | -0.87 | 0.97 | 0.001 |
| 12 | 4,4-Dimethyl-cyclohex-2-en-1-ol | -0.21 | -0.98 | 0.70 | 0.009 |
| 13 | Borane, ethylisopropylmethyl- | -0.31 | -0.95 | 0.90 | 0.001 |
| 14 | Dill ether | -0.69 | -0.72 | 0.97 | 0.001 |
| 15 | cis,cis-Nepetalactone | -0.66 | 0.75 | 0.99 | 0.002 |
| 16 | cis-trans-Nepetalactone | -0.66 | 0.75 | 0.99 | 0.002 |
| 17 | (+)-Isodihydronepetalactone | -1.00 | -0.09 | 0.99 | 0.001 |
| 18 | Iridomyrmecin | -0.95 | 0.32 | 0.89 | 0.001 |
| 19 | (+)-Dihydronepetalactone | -0.96 | 0.30 | 0.87 | 0.001 |
| 20 | 3-Oxabicyclo[5.3.0]decan-2-one, 9-methylene-, trans- | -0.67 | -0.74 | 0.86 | 0.004 |
|  |  |  |  |  |  |
| **b. Solvent extraction** | |  |  |  |  |
| 21 | Actinidine | 0.99 | -0.17 | 0.91 | 0.001 |
| 22 | cis,cis-Nepetalactone | 0.21 | 0.98 | 0.35 | 0.144 |
| 23 | (+)-Isodihydronepetalactone | 0.89 | 0.45 | 0.99 | 0.001 |
| 24 | (+)-Dihydronepetalactone | 0.96 | 0.26 | 0.98 | 0.001 |
| 25 | 2H-Oxecin-2-one, 3,4,7,8,9,10-hexahydro-4-hydroxy-10-methyl-, [4S-(4R*,5E,10S*)]- | 0.99 | -0.14 | 0.96 | 0.001 |
| 26 | 3-Oxabicyclo[5.3.0]decan-2-one, 9-methylene-, trans- | 0.91 | 0.41 | 0.85 | 0.001 |
| 27 | Palmitic acid | 0.86 | -0.51 | 0.84 | 0.003 |
| 28 | Phenol, 2,4-bis(1-methyl-1-phenylethyl)-RI2508 | 0.34 | -0.94 | 0.96 | 0.001 |
| 29 | Unknown-RT49.05 | 0.05 | 1.00 | 0.94 | 0.001 |
